# Exploring glycine root uptake dynamics in phosphorus and iron deficient tomato plants during the initial stages of plant development

**DOI:** 10.1186/s12870-024-05120-6

**Published:** 2024-06-03

**Authors:** F. Trevisan, F. Waschgler, R. Tiziani, S. Cesco, T. Mimmo

**Affiliations:** 1 Faculty of Agricultural, Environmental and Food Sciences, Free University of Bolzano, Bolzano, 39100 Italy; 2 Competence Centre for Plant Health, Free University of Bolzano, Bolzano, 39100 Italy

**Keywords:** Phosphorus, Iron, Nutrient deficiency, Tomato (*Solanum lycopersicum* L.), Root exudates acquisition, Glycine uptake

## Abstract

**Background:**

Phosphorus (P) and iron (Fe) deficiencies are relevant plants nutritional disorders, prompting responses such as increased root exudation to aid nutrient uptake, albeit at an energy cost. Reacquiring and reusing exudates could represent an efficient energy and nitrogen saving strategy. Hence, we investigated the impact of plant development, Fe and P deficiencies on this process.

Tomato seedlings were grown hydroponically for 3 weeks in Control, -Fe, and -P conditions and sampled twice a week. We used Isotope Ratio Mass-Spectrometry to measure δ^13^C in roots and shoots after a 2-h exposure to ^13^C-labeled glycine (0, 50, or 500 μmol L^−1^). Plant physiology was assessed with an InfraRed Gas Analyzer and ionome with an Inductively Coupled Plasma Mass-Spectrometry.

**Results:**

Glycine uptake varied with concentration, suggesting an involvement of root transporters with different substrate affinities. The uptake decreased over time, with -Fe and -P showing significantly higher values as compared to the Control. This highlights its importance during germination and in nutrient-deficient plants. Translocation to shoots declined over time in -P and Control but increased in -Fe plants, suggesting a role of Gly in the Fe xylem transport.

**Conclusions:**

Root exudates, *i.e.* glycine, acquisition and their subsequent shoot translocation depend on Fe and P deficiency. The present findings highlight the importance of this adaptation to nutrient deficiencies, that can potentially enhance plants fitness. A thorough comprehension of this trait holds potential significance for selecting cultivars that can better withstand abiotic stresses.

**Supplementary Information:**

The online version contains supplementary material available at 10.1186/s12870-024-05120-6.

## Background

Nutritional deficiencies, such as phosphorus (P) and iron (Fe), are among the most limiting factors for agricultural production [1]. To meet the still growing food demand, there is urgency to achieve a sustainable intensification of global food production. It is therefore essential to comprehend how crops interact with their environment, with particular emphasis on the rhizosphere from which they retrieve most of their resources. Plants shape and interact with the rhizosphere through the secretion of multiple root exudates. Root exudates play pivotal roles in influencing soil microbial community [2–4], soil structure [5, 6], nutrient availability and mobilization processes [7], toxicity of metals [8, 9] and plant defence mechanisms against pathogens [10, 11]. The multiple functions of root exudates in the rhizosphere are complemented by an equally large chemical variability. Indeed, plants may release amino acids, organic acids, carbohydrates (primary metabolites), as well as phenolic compounds, plant hormones, and glucosinolates (secondary metabolites) through the roots [12].

Despite the large diversity of exudates, glycine (Gly), together with serine and alanine, is one of the 3 major amino acids released by roots [13, 14]. Glycine can chelate Fe ions (logK [Fe(III)Gly] = 8.57–9.25; logK [Fe(II)Gly] = 4.13–4.36 [15, 16]. Complexing Fe can directly enhance the availability of Fe and indirectly phosphorus (P) in the rhizosphere, thereby rendering them more readily accessible for plant uptake. For instance, Gly chelates Fe from FePO_4_ or Fe_3_(PO_4_)_2_, forming Fe-Gly and Fe-bisGly complexes, and therefore solubilizing PO_4_^3−^ in form of H_2_PO_4_^−^ or HPO_4_^2−^ which will otherwise be insoluble [17]. Glycine can further affect the redox state of Fe in the soil, leading to reduction of Fe(III) in Fe(II), the most soluble and bioavailable form [17–19]. Moreover, microbial activities stimulated by Gly can in turn impact nutrient cycling and availability in the rhizosphere [20, 21]. Lastly, the importance of Gly as organic N supply, *i.e.* as fertilizer, in agricultural and forest ecosystems was repeatedly proven [22, 23]. Glycine concentration in soil solution was found to be around 10 μmol L^−1^, but most likely much higher in the rhizosphere [23, 24].

Exudates constitutes a significant energy investment for crops [25, 26], which is even greater considering the energy used for their biosynthesis and exudation [27]. Consequently, the possibility of plants to reacquire exudates from the rhizosphere represents an intriguing energy (C) and nitrogen (N) saving strategy. This concept has been hypothesized [26] and demonstrated only recently [28–30]. For example, both wheat and tomato plants can exude and reuptake various organic compounds from the rhizosphere. The uptake appears to be particularly enhanced under P deficiency, supporting the idea that plants may reclaim exudates to save carbon, N and therefore energy [30]. Despite this topic holds potential significance for selecting cultivars that can better withstand biotic and abiotic stresses, the literature contains relatively few studies on the uptake of root exudates. Hence, we aimed at deciphering whether the uptake and the consequent shoot translocation of the acquired root exudates in tomato plants, are altered by the plants nutritional and physio-phenological developmental state. To answer this research question 1-week-old tomato plants (*Solanum lycopersicum* L.) were grown hydroponically under P or Fe deficiencies for further 17 days. Every 3rd day, after feeding the plants with ^13^C-labeled Gly for 2 h, roots were separated from shoots and their ^13^C content was analysed while monitoring plants physiological and nutritional state.

## Methods

### Growing conditions

Seeds of tomato (*Solanum lycopersicum* L. cv. Marmande) were germinated on moistened tissue paper (0.5 mmol L^−1^ CaSO_4_) in darkness at 25 °C for 7 days [31]. The germinated seedlings were transferred into plastic pots, with each pot accommodating 10 seedlings. Each pot contained 1.5 L of aerated nutrient solution (NS) according to [32], with the following composition mmol L^−1^: 2 Ca(NO_3_)_2_, 0.7 K_2_SO_4_, 0.1 KH_2_PO_4_, 0.1 KCl, 0.5 MgSO_4_; and μmol L^−1^: 10 H_3_BO_3_, 0.5 MnSO_4_, 0.2 CuSO_4_, 0.5 ZnSO_4_, 0.01 (NH_4_)_6_Mo_7_O_24_, and 80 Fe(III)-EDTA. Every 3 days the NS was renewed. After 7 days of growth in the complete NS, two-thirds of the plants were relocated to treatment-specific NS solutions for an additional 17 days. The remaining one-third was maintained in the complete NS as a Control group. Specifically, the treatments involved subjecting one-third of the plants to phosphorus deficiency (-P) and one-third to iron deficiency (-Fe). Each treatment group consisted of five biological replicates for each time point. Throughout the entire growth period (24 days), the environmental conditions within the growth chamber were maintained constant: 14:10 light:darkness, a temperature regimen of 24 °C:19 °C, and a relative humidity of 70%.

### Analysis of physiological parameters

On the final sampling day, *i.e.* 17 days after treatment (DAT), just before the plants were harvested, the leaf CO_2_ and water vapour fluxes were assessed with an InfraRed Gas Analyzer (IRGA) equipped with a small leaf chamber (2.16 cm^2^) (ACD BioScientific Ltd. – Lcpro T). Photosynthetic rate, stomatal conductance, and transpiration rate were calculated starting from the directly measured parameters, namely, CO_2_ concentration and relative humidity from beneath the leaf and from the reference probe far from any CO_2_ or H_2_O source, leaf temperature, Photosynthetic Active Radiation (PAR) at leaf level, atmospheric pressure, and effective molar air flow. Multiple measurements were taken from fully expanded 17 days old leaves on various biological replicates for each treatment (*n* ≥ 9).

### Glycine uptake

Glycine uptake and subsequent sampling occurred twice a week over three consecutive weeks, totalling six samplings on 0, 3, 7, 10, 14, and 17 DAT. To maintain root integrity, the plants were delicately removed from their pots and immersed in a 0.5 mmol L^−1^ CaSO_4_ solution for 15 minutes [33]. After this recovery step, two-thirds of the plants were transferred to either a 50 μmol L^−1^, or 500 μmol L^−1^ aerated ^13^C-labeled Gly solution for 2 h. The ^13^C-labeled Gly was labeled in the carboxylic carbon with 99 atom % ^13^C. Glycine concentration was selected according to literature, indeed in soil solution Gly was found to be around 10 μmol L^−1^, but most likely much higher in the rhizosphere [23, 24]. The remaining one-third of the plants were moved into Milli-Q water representing the Control treatment. All solutions were supplemented with 1 mg L^−1^ of Micropur (Katadyn) to prevent microbial growth and Gly degradation during the experiment. Following the 2-h exposure, the roots underwent five thorough sequential washes with Milli-Q water. The plants were dried with tissue paper, the shoots were separated from the roots and individually weighed (fresh weight (FW)). The plant samples were then dried in an oven at 70 °C until constant weight was reached (dry weight (DW)). The dried samples were ball-milled (Mixer Mill MM 400, Retsch, Italy) for 1 min at 30 Hz.

### Carbon isotope ratio determination

Approx. 0.25 mg of the dried and homogenized samples were carefully weighted into tin capsules. The samples underwent complete combustion prior to the determination of δ^13^C by an Elemental Analyzer (EA Flash 1112 Thermo Scientific, Germany) coupled with a Continuous Flow Isotope Ratio Mass Spectrometer (IRMS, Delta V Thermo Scientific, Germany). Under the operating conditions, the oxidation furnace was set to 1020°C, and the reduction furnace was maintained at 900°C. H_2_O was eliminated using a Mg(ClO_4_)_2_ trap. The stable carbon isotope ratios are expressed in δ‰ relative to an international reference, namely V-PDB (Vienna–Pee Dee Belemnite), calculated using the following formula:

1 $$\delta^{13}C{\permil}=\frac{(R_{sample}-R_{standard})}{R_{standard}}\times10^3$$

where R represents the ratio between the heavier and lighter isotopes. R_sample_ denotes the carbon isotope ratio determined for the specific sample, whether it be root or shoot, while R_standard_ represents the carbon isotope ratio of a reference material, namely Vienna – Pee Dee Belemnite [34, 35].

To ensure the analytical accuracy and reliability of the results, four sets of three working standards, specifically IAEA 600 (caffeine), IAEA CH_3_ (cellulose), and acetanilide, were subjected to analysis at the beginning, midway through, and conclusion of the analysis. The margin of uncertainty associated with the determination of carbon isotopes was ± 0.2‰.

Starting from the determined δ^13^C, the isotopic mass balance, representing the “mg” of Gly taken up per “g” of tissue DW, was computed through the following formula [30, 36]:

2 $${f}_{r}\left[\frac{mg [Gly]}{g [DW]}\right]= \frac{{\delta }^{13}{C}_{TP}-{\delta }^{13}{C}_{C}}{{\delta }^{13}{C}_{CS}-{\delta }^{13}{C}_{C}}\times \% C\times {10}^{3}$$

where δ_CTP_ represents the δ^13^C value for plants exposed to either 50 µmol L^−1^ or 500 µmol L^−1^ of ^13^C-labeled Gly. δ^13^C_C_ denotes the δ^13^C value for plants treated with 0 µmol L^−1^ of Gly (MilliQ water). δ^13^C_CS_ stands for the δ^13^C value of the ^13^C-labeled Gly itself. %C refers to the carbon content as percentage of the DW.

### Elemental analysis

Approx. 0.25 g of each sample were acid digested with concentrated ultrapure HNO_3_ (69% w/w) using a single reaction chamber microwave digestion system (UltraWAVE, Milestone, Shelton, CT, United States). Macro- and micronutrient concentrations were then determined by Inductively Coupled Plasma-Mass Spectrometer (ICP-MS) (Agilent 7800 ICP-MS, United States), using La as an internal standard and tomato leaves (SRM 1573a) as external certified reference material.

### Statistical analysis

The statistical analysis of the raw data was conducted using R × 64 version 4.2.3 software. To aid in this analysis, specific R packages were employed: agricolae, which facilitated statistical analysis [37], and ggplot2, which aided in visualizing the data [38]. All results are presented as means accompanied by their standard errors. The assumptions of normality and homogeneity of variance were tested with the Shapiro–Wilk and Levene test respectively. To assess the differences among various treatments, multiple comparisons were executed using two-way analysis of variance (ANOVA) and one-way ANOVA, followed by Tukey’s post hoc test, with a *p*-value < 0.05. Further, multiple linear models were calculated with their 95% Confidence Intervals (CI). The analysis of covariance (ANCOVA) followed by Tukey’s post hoc test, with a *p*-value < 0.05, was performed to compare the slopes of the different linear models.

## Results

### Elemental analysis

**Fig. 1:**
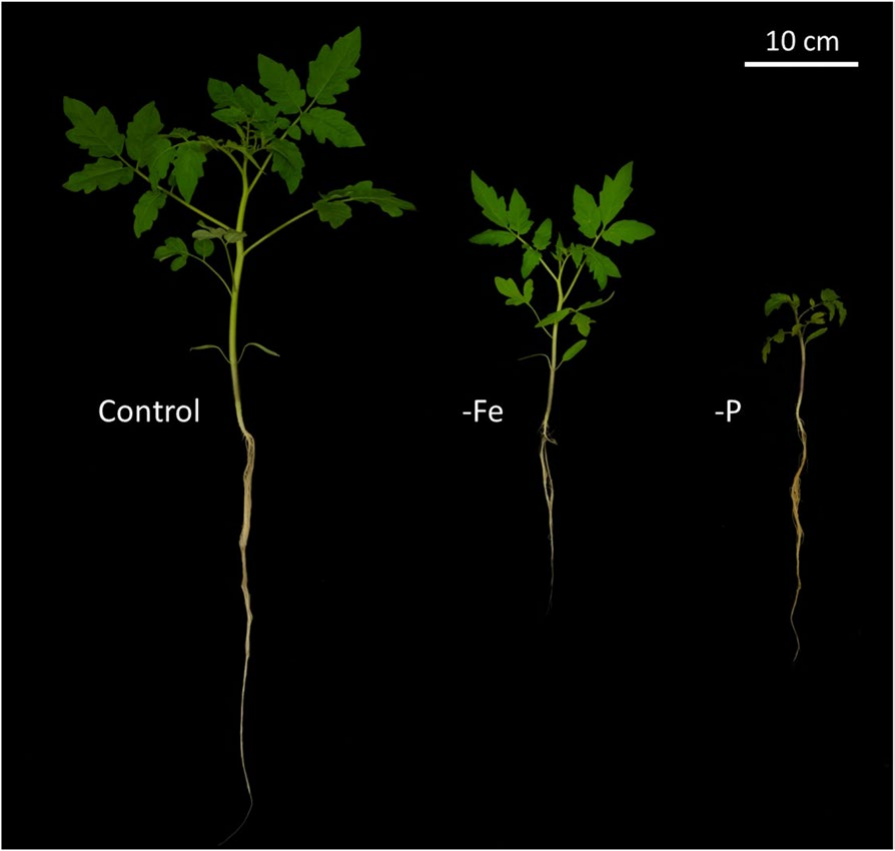
Picture of tomato (*Solanum lycopersicum* L.) plants at harvest (*i.e.* 17 DAT). Control conditions (Control), phosphorus deficiency (-P) and iron deficiency (-Fe). DAT = Days After Treatment

**Fig. 2:**
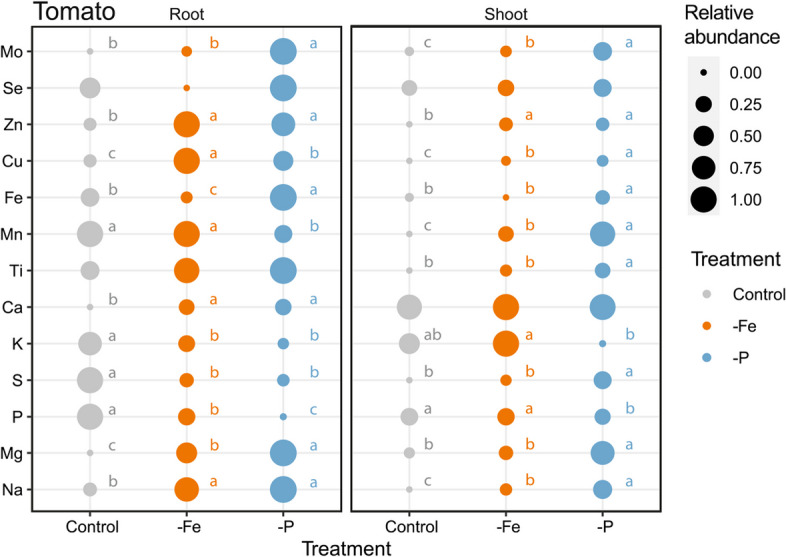
Bubble plot of the elemental composition of tomato (*Solanum lycopersicum* L.) plants grown under control conditions (Control), phosphorus starvation (-P) and iron starvation (-Fe) plotted over time with *n* = 5 for shoots and *n* = 4 roots. Letters next to the boxes indicate statistically significant differences (*p* < 0.05) between treatments assessed by a one-way ANOVA with Tukey post hoc test

Tomato elemental profile was assessed in both shoots and roots at harvest (*i.e.* 17 DAT). The One-Way-ANOVA showed a significantly different accumulation of mineral elements in response to the treatments (Fig. 2 & Table 1, S1). The nutrient deficiencies set as treatments were confirmed: -P plants had lower P concentration as compared to Control (-88% in roots; -24% in shoots) and -Fe (-69% in roots; -21% in shoots). Similarly, the Fe concentration in -Fe roots was lower as compared to Control (-75%) and -P (-90%) but in -Fe shoots the Fe content was only lower than -P (-85%) and equal to the Control (-38% n.s.) (Fig. 1). Besides this proof of the experimental setup, nutrient deficient plants, -Fe and especially -P, showed a significant accumulation of the mineral nutrients as compared to the Control in both under- and above-ground tissues. More precisely, -P plants acquired and stored higher amounts of Mo, Zn, Cu, Fe, Mg and Na in both roots and shoots, Mn and S concentrations were significantly higher in shoots, while Ca was significantly higher in roots. Similarly, -Fe plants acquired and stored higher amounts of Zn, Cu and Na in both roots and shoots, Mo and Mn concentrations were significantly higher in shoots, while Ca and Mg were significantly higher in roots (Fig. 2 & Table 1).

**Table 1:** Table showing the elemental composition of tomato (*Solanum lycopersicum* L.) plants grown under control conditions (Control), phosphorus starvation (-P) and iron starvation (-Fe). The data are expressed as mean ± standard error. *N* = number of biological replicates. The letters indicate statistically significant differences (*p* < 0.05) between treatments assessed by a one-way ANOVA with Tukey post hoc test

Tissue	Treatment	N	Na (mg g^-1^ DW)	Mg (mg g^-1^ DW)	P (mg g^-1^ DW)	S (mg g^-1^ DW)	K (mg g^-1^ DW)	Ca (mg g^-1^ DW)	Fe (mg g^-1^ DW)	Zn (mg g^-1^ DW)	Mn (µg g^-1^ DW)	Cu (µg g^-1^ DW)	Mo (µg g^-1^ DW)	Ti (µg g^-1^ DW)	Se (µg g^-1^ DW)
*Root*	Control	4	1.658 ± 0.099 b	4.629 ± 0.091 c	16.204 ± 0.291 a	23.040 ± 0.190 a	58.793 ± 1.397 a	6.641 ± 0.111 b	1.528 ± 0.105 b	0.161 ± 0.012 b	221.950 ± 16.027 a	25.400 ± 0.743 c	1.947 ± 0.145 b	1.583 ± 0.126	1.228 ± 0.490
	-Fe	4	8.309 ± 1.530 a	9.485 ± 0.419 b	6.214 ± 0.442 b	11.912 ± 0.804 b	50.301 ± 2.988 b	13.191 ± 1.216 a	0.389 ± 0.051 c	1.181 ± 0.149 a	220.043 ± 26.243 a	154.702 ± 16.346 a	3.234 ± 0.342 b	2.923 ± 0.669	0.358 ± 0.330
	-P	4	9.898 ± 1.216 a	13.634 ± 0.459 a	1.946 ± 0.058 c	10.849 ± 0.492 b	46.256 ± 1.310 b	13.370 ± 0.833 a	3.860 ± 0.141 a	0.863 ± 0.071 a	98.844 ± 9.036 b	71.572 ± 6.465 b	31.462 ± 5.069 a	3.143 ± 0.546	1.999 ± 0.851
*Shoot*	Control	5	0.318 ± 0.020 c	5.136 ± 0.094 b	6.547 ± 0.200 a	9.822 ± 0.442 b	54.948 ± 1.243 ab	33.020 ± 0.386	0.163 ± 0.005 b	0.037 ± 0.003 b	43.779 ± 0.648 c	8.526 ± 0.335 c	2.587 ± 0.243 c	0.572 ± 0.042 b	0.721 ± 0.271
	-Fe	5	1.167 ± 0.033 b	6.083 ± 0.364 b	6.321 ± 0.474 a	10.594 ± 0.283 b	63.270 ± 6.127 a	36.123 ± 2.385	0.100 ± 0.029 b	0.195 ± 0.020 a	82.121 ± 12.148 b	12.915 ± 1.082 b	3.939 ± 0.226 b	0.780 ± 0.059 b	0.782 ± 0.356
	-P	4	3.874 ± 0.443 a	11.222 ± 0.319 a	5.005 ± 0.135 b	13.900 ± 0.297 a	45.373 ± 0.328 b	33.580 ± 0.839	0.648 ± 0.030 a	0.157 ± 0.027 a	193.998 ± 6.424 a	16.790 ± 0.519 a	12.322 ± 0.225 a	1.059 ± 0.070 a	0.868 ± 0.095

### Physiological parameters

**Fig. 3:**
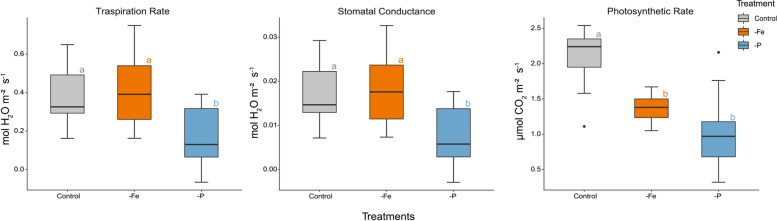
Photosynthetic rate (µmol CO_2_ m^-2^ s^-1^), transpiration rate and leaf stomatal conductance (mmol H_2_O m^-2^ s^-1^) of tomato (*Solanum lycopersicum* L.) plants according to three different treatments: Control (Control), phosphorus deficiency (-P) and iron deficiency (-Fe) with *n* ≥ 9. Letters next to the boxes indicate statistically significant differences (*p* < 0.05) between treatments assessed by a one-way ANOVA with Tukey post hoc test

Tomato physiological state was assessed right before harvest (*i.e.* 17 DAT). The One-Way-ANOVA showed statistically significant treatment derived differences in transpiration rate, stomatal conductance and photosynthetic rate (Fig. 3 & Table S2). The photosynthetic rate was significantly higher in Control plants as compared to both Fe and P deficient plants (+ 52% and + 100%, respectively), while there was no difference between Fe and P deficient plants (Fig. 3). On the contrary, both transpiration rate and stomatal conductance were significantly higher in Control (+ 133% and + 137%, respectively) and -Fe (+ 145% and + 145%, respectively) plants as compared to the -P which had a much lower transpiration rate and stomatal conductance (Fig. 3).

### Mass balance of glycine uptake

**Fig. 4:**
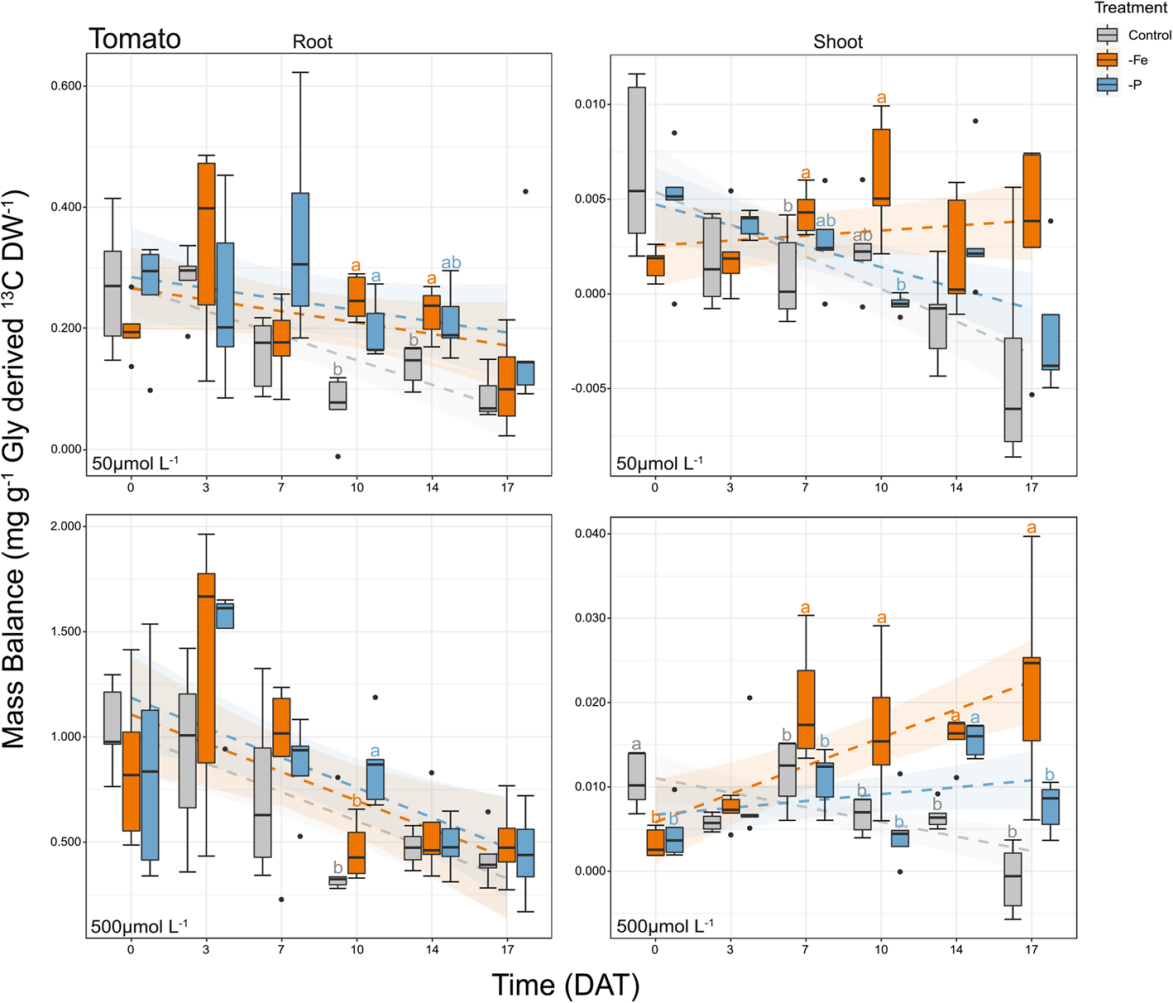
Mass balance (mg g^-1^ Gly derived ^13^C DW^-1^) of tomato (*Solanum lycopersicum* L.) plants grown under control conditions (Control), phosphorus starvation (-P) and iron starvation (-Fe) plotted over time with *n* = 5. Day 0 on the x-axis indicates the day on which some of the plants were transferred to the treatment-specific nutrient solutions (NSs), that is, after 7 days of growth in full NS. The graphs on the left show the mass balance values of roots, while the ones on the right the mass balance of the shoots. The top two graphs refer to the tomato plants exposed to ^13^C Gly at 50 μmol L^-1^ while the bottom two to ^13^C Gly at 500 μmol L^-1^ as reported in the bottom left of each graph. The semi-transparent halo around each trendline represents the 95% confidence interval of the trendline. Letters next to the boxes indicate statistically significant differences (*p* < 0.05) between treatments assessed by a one-way ANOVA with Tukey post hoc test. DW = Dry Weight; DAT = Days After Treatment

The mass balance was calculated starting from the δ^13^C values of tomato roots and shoots determined by the IRMS every third day, *i.e.* on 0, 3, 7, 10, 14, and 17 DAT. Figure 4, which summarize the mass balance results, shows how 95–99% of the Gly derived ^13^C remains in roots after uptake. Moreover, the two-way-ANOVA performed on the mass balance data showed a significant effect of time, namely of the plant physio-phenological developmental state, on the uptake of Gly by roots and on its translocation to shoots in plants treated with either the 50 or 500 μmol L^−1 13^C-labeled Gly solution (Fig. 4 & Table S3). On the contrary, the effect of treatments, namely of the plant nutritional state, was significant only in roots exposed to the 50 μmol L^−1 13^C-labeled Gly solution and in shoots of plants which roots were exposed to either the 50 or 500 μmol L^−1 13^C-labeled Gly solution (Fig. 4). Moreover, the two-way-ANOVA highlighted a significant interaction between treatments and time in the same three conditions: roots exposed to the 50 μmol L^−1 13^C-labeled Gly solution and in shoots of plants which roots were exposed to either the 50 or 500 μmol L^−1 13^C-labeled Gly solution (Fig. 4). This result indicates that in these conditions the effect of the treatments, *i.e.* nutrient deficiencies, varied over time. More specifically the differences between treatments and Control increased during plant development.

The fitted regression models show an overall decreasing Gly uptake over time especially when roots are exposed to the highest concentration of ^13^C-labeled Gly (Fig. 4 & Table S4). Despite this general trend, in -P and -Fe plants exposed to the 50 μmol L^−1 13^C-labeled Gly solution the uptake remained constant over time, and significantly higher as compared to the Control (on average + 115% and + 104%, in -P and -Fe respectively) (Fig. 4 & Table S3, S4). Similarly, the translocation of Gly derived ^13^C decreased in Control plants exposed to both high and low concentrations of ^13^C-labeled Gly solution and in -P plants exposed to the 50 μmol L^−1 13^C-labeled Gly solution. On the contrary, the translocation of Gly derived ^13^C in Fe deficient plants remained constant over time (significantly higher than the Control but the same as -P) when the plants were exposed to the low concentration of ^13^C-labeled Gly solution. The translocation of Gly derived ^13^C in Fe deficient plants even increased over time when the plants were exposed to the high concentration of ^13^C-labeled Gly solution (significantly higher than both -P and Control). In -P plants, instead, the translocation remained constant over time at the same Gly concentration (Fig. 4).

## Discussion

Nutrient deficiencies can induce changes in the morphology and physiology of plants (Figs. 1 and 3) [31, 39]. Within these alterations, root exudation represents one of the most important processes to improve nutrient acquisition and to enhance plants´ chances of survival [7, 40]. However, despite the importance of these root exudates in the rhizosphere, only little is known about their uptake and possible reutilization, especially under nutrient deficiencies where a reacquisition might represent a crucial energy and N saving strategy. Our study investigated therefore the acquisition of Gly, identified as a major root exudate, both in terms of concentration and allocation in tomato plants subjected to Fe and P deficiency [18, 21, 41].

The assessment of the morphological and physiological state (Figs. 1 and 3) of tomato plants shows that both -Fe and -P plants have a stunted growth (Fig. S1 & Table S5) and reduced photosynthetic activity similarly to what is already reported in literature [31, 42]. The ICP-MS analysis highlighted how the ionomic profile of both roots and shoots collected from plants subjected to the two nutritional deficiencies here studied, was impaired as compared to the Control. The elemental composition (Fig. 2 & Table 1) of the tomato tissues confirmed the reliability of the nutrient deficiencies set as treatments, showing significantly lower accumulation of P and Fe in -P and -Fe conditions, respectively. Moreover, -P and -Fe compared to Control plants, tend to have significantly higher levels of other micro- and macro nutrients similarly to what described by Tiziani et al. [31] in tomato and highlighted in cucumber by Tomasi et al. [43]. This is claimed to be related to a biomass *dilution effect* as well as to a functional compensation and an imbalanced ion-uptake [44–46]. Indeed, the increase in biomass observed in developing seedlings could determine a decrease in concentration (*dilution*) of multiple nutrients [44], which content is considerably higher in seeds as compared to adult plants [47]. Consequently, the stunted growth of a -P plant as compared to a Control plant, might decrease this nutrient dilution effect, actually increasing the concentration of certain elements in the biomass of young seedlings. This theory and the mechanisms behind it were thoroughly investigated in the 70s-80s of the past century but it seems to have been partially forgotten since recent literature does not mention them. Alternatively, plants may substitute one limiting element for another to achieve functional compensation. For instance, -P plants, might acquire higher amounts of sulphur to substitute phospholipids with sulfolipids [46, 48]. Furthermore, plants perceiving Fe deficiency take up higher amounts of Zn, Cu, Mn (Fig. 2 & Table 1), as a side effect of the upregulation of IRT1 transporter and of the increased release of organic acids that facilitate the uptake not only of Fe but also of other metals [45, 49, 50].

The IRMS analysis confirmed previous results on the altered ^13^C discrimination by tomato plants exposed to different nutrient limitations (Fig. S2 & Table S6) [30, 42]. The mass balance data (Fig. 4), show that Gly was taken up by tomato plants and that the great majority (95–99%) of the Gly derived ^13^C is stored and/or metabolized in the root while only a minor proportion (1–5%) is translocated to shoots as seen also by Warren [51]. This finding was consistent across all treatments, *i.e.* Control, -P and -Fe. This highlights the importance of Gly at root level for nutrient complexation and acquisition [52, 53]. We further hypothesised that this Gly root accumulation might be important for the biosynthesis of metabolites like glutathione, which is essential for preventing oxidative stress, especially in plants facing abiotic stresses [54]. Moreover, the taken up Gly might be utilized at root level, for the biosynthesis of Gly-rich proteins (GRP) which are involved in cellular stress responses and signalling [55]. These results are in accordance with literature pointing at roots as main sink tissue [56]. Indeed, the flow of carbon, in developing seedlings is mainly from shoots to roots and not vice versa, which agrees with the present results showing that the Gly derived ^13^C remains for a large proportion in roots. Caution should however be paid in hypothesizing possible roles of Gly in roots and shoots since the present study performed a bulk stable isotope analysis. Indeed, the ^13^C derived from Gly might be incorporated in several different metabolites [51, 57].

In all treatments, the time-course experiment shows how Gly uptake tends to decrease over time (between 0 and 17 DAT) (Fig. 4). This suggests that the acquisition of exudates as an energy and N saving strategy might be particularly important in roots of developing seedlings, *i.e.* during the initial phases of plants development. These results relate well with previous knowledge showing that exudation decreases with plant age [58, 59].

The plants nutritional state significantly altered Gly root uptake. Indeed, P and Fe deficient plants took up significantly more Gly as compared to the Control (on average + 115% and + 104%, respectively) (Table S3). These are the first pieces of evidence ever that plants facing Fe deficiency not only take up Gly, but take up more Gly than Control plants. A possible explanation of the increased Gly uptake in -Fe plants might be ascribed to a N uptake compensation for the well-known depression of root nitrate uptake in Fe-deficient dicotyledonous species [43]. The effect of nutrient deficiencies on the uptake was significant only in plants exposed to the 50 μmol L^−1 13^C-labeled Gly solution (Fig. 4). This indicates that the impact of nutrient deficiency on the uptake depends on Gly concentration. This might suggest that root Gly transporters, *e.g.* from the amino acid permease (AAP) family, with different substrate affinities are regulated differently according to the plants nutritional state [60, 61]. More precisely it is hypothesized that low-capacity high-affinity Gly transporters, which are responsible for the uptake of Gly at low concentrations (50 μmol L^−1^), are upregulated when the plant is facing Fe and P deficiencies. On the contrary, the regulation of high-capacity low-affinity Gly transports, responsible for the uptake of Gly at higher concentration (500 μmol L^−1^), remained unchanged under nutrient deficiencies as it was seen for nitrate [62]. However, the opportunity that the higher Gly concentration (500 µmol L^−1^) saturates Gly transporters, masking the impact of nutrient deficiencies on the uptake of Gly cannot be excluded. The translocation of Gly derived ^13^C from roots to shoots remained instead constant over time in Control and -P plants, while it increased in -Fe plants (Fig. 4). The increased transport of Gly derived ^13^C to shoots in -Fe plants was significant in plants exposed to both to 50 μmol L^−1^ and 500 μmol L^−1 13^C-labeled Gly solution. Considering both the Gly mobility in the xylem [63] and the possibility of Gly to form Gly Fe-complexes [15–17], an involvement of this amino acid in the xylem Fe translocation to shoots cannot be excluded. This hypothesis might explain in fact why Gly derived ^13^C was higher in -Fe shoots as compared to -P and Control shoots. Moreover, this observed increase in Gly derived ^13^C translocation to shoots in -Fe plants indicates that -Fe shoots might be a main sink tissue. This is in contrast with literature showing that in later developmental stages shoots lose importance as sink tissues [56].

## Conclusions

The results of the present work show that Gly can be taken up by tomato roots and that this uptake is enhanced when plants are suffering Fe and P deficiency. Gly uptake by roots decreases over time, highlighting the importance of this energy and N saving strategy in the first phenological plant stages, namely at germination, when the needs of nutrients and C are more pronounced, particularly in roots. Once acquired, the majority of Gly remained in roots, while only a limited proportion was translocated to shoots, underlining the sink role of the root tissue after germination. Similarly to the uptake, also the translocation of Gly derived ^13^C to shoots decreased over time for Control and -P plants. On the contrary, the translocation of Gly derived ^13^C to shoots increased with plant age when plants are exposed to Fe shortage, suggesting a possible involvement of Gly in the Fe xylem transport, N compensation or more in general in the Fe plant homeostasis. In addition, the differences seen between the high (500 µmol L^−1^) and low (50 µmol L^−1^) Gly concentrations might be ascribed to different root Gly transporters as well as due to a saturation of the latter. In conclusion, our results provide evidence that the acquisition of root exudates like Gly, can represent an important adaptation to nutrient deficiencies potentially enhancing plants fitness. A better understanding of this plant trait holds potential significance for selecting cultivars that can better withstand abiotic stresses.

### Supplementary Information

Supplementary Material 1.

Supplementary Material 2.

Supplementary Material 3.

Supplementary Material 4.

Supplementary Material 5.

Supplementary Material 6.

Supplementary Material 7.

## Data Availability

The following information was supplied regarding data and code availability: the raw data, the version of the individual packages and scripts used to analyse the data and generate the figures of this study are available at GitHub: https://github.com/Fabio-Trevisan/Gly_Ruptake.

## Abbreviations

ANCOVA: Analysis of covariance
ANOVA: Analysis of variance
C: Carbon
DAT: Days after treatment
DW: Dry weight
Fe: Iron
FW: Fresh weight
Gly: Glycine
ICP-MS: Inductively Coupled Plasma-Mass Spectrometer
IRGA: Infra-red gas analyser
IRMS: Isotope Ratio Mass Spectrometer
N: Nitrogen
n.s.: Not significant
NS: Nutrient solution
P: Phosphorus
δ^13^C: Stable carbon isotope ratio
