# Supplementary material for: Exploring glycine root uptake dynamics in phosphorus and iron deficient tomato plants during the initial stages of plant development
Source: BMC Plant Biol. 2024 Jun 3;24:495. doi: 10.1186/s12870-024-05120-6 (PMC11145798; doi:10.1186/s12870-024-05120-6)
Supplement: Supplementary file 1 — Supplementary Material 1. [file 12870_2024_5120_MOESM1_ESM.pdf]

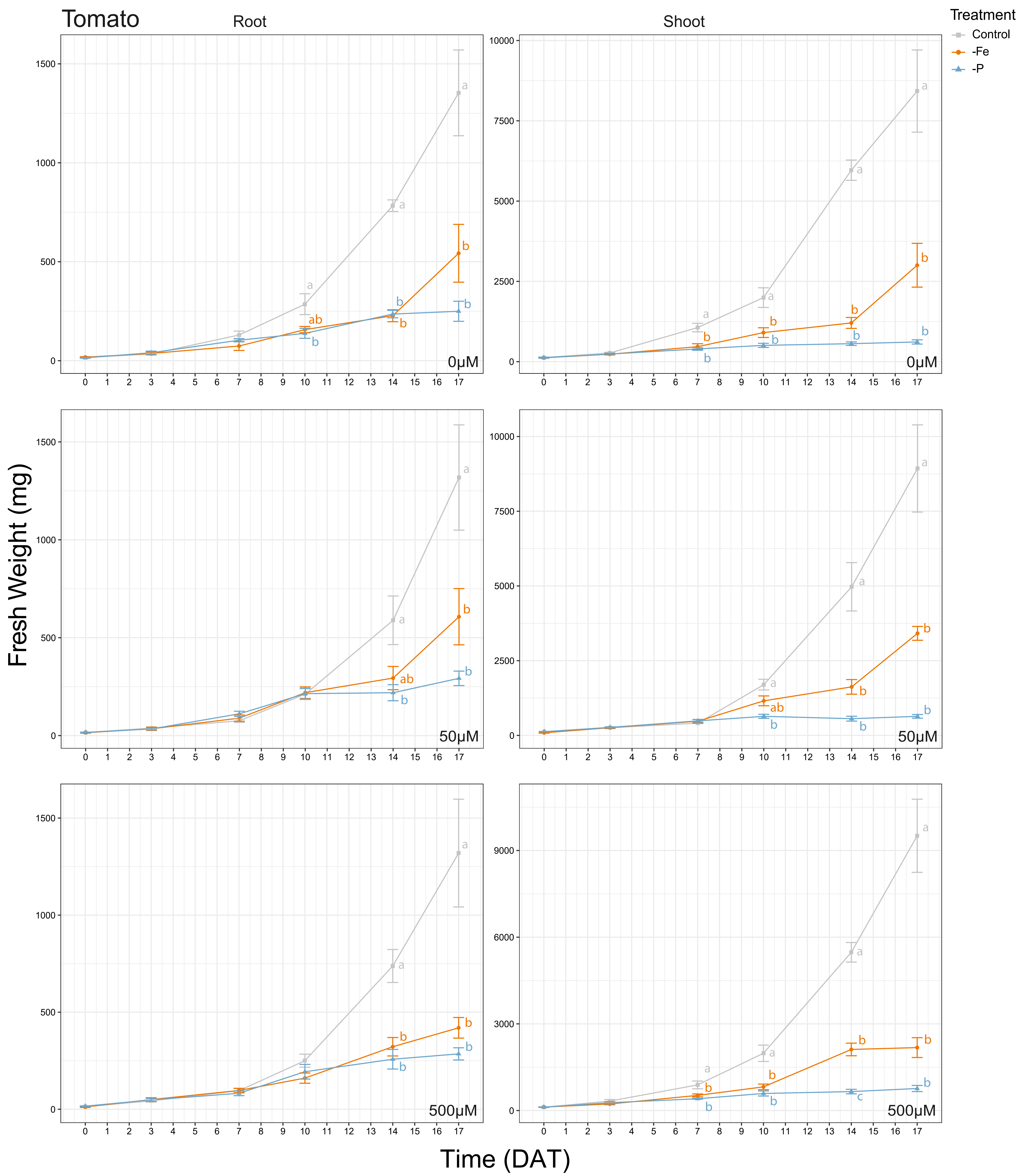

Figure S1: Fresh weight (mg) of tomato (*Solanum lycopersicon* L.) plants grown under control conditions (Control), phosphorus starvation (-P) and iron starvation (-Fe) plotted over time. Day 0 on the x-axis indicates the day on which some of the plants were transferred to the treatment-specific nutrient solutions (NSs), that is, after 7 days of growth in full NS. The fresh weights of roots, left, and shoots, right, are represented as mean ± standard error, n = 7. The top two graphs refer to tomato plants exposed to ^{13}C Gly at 0 μmol L^{-1} (Millli-Q water), the middle two graphs to ^{13}C Gly at 50 μmol L^{-1}, while the bottom two to ^{13}C Gly at 500 μmol L^{-1} as reported in the bottom left of each graph. Letters next to the points indicate statistically significant differences ($p < 0.05$) between treatments assessed by a one-way ANOVA with Tukey post hoc test. DAT = Days After Treatment.

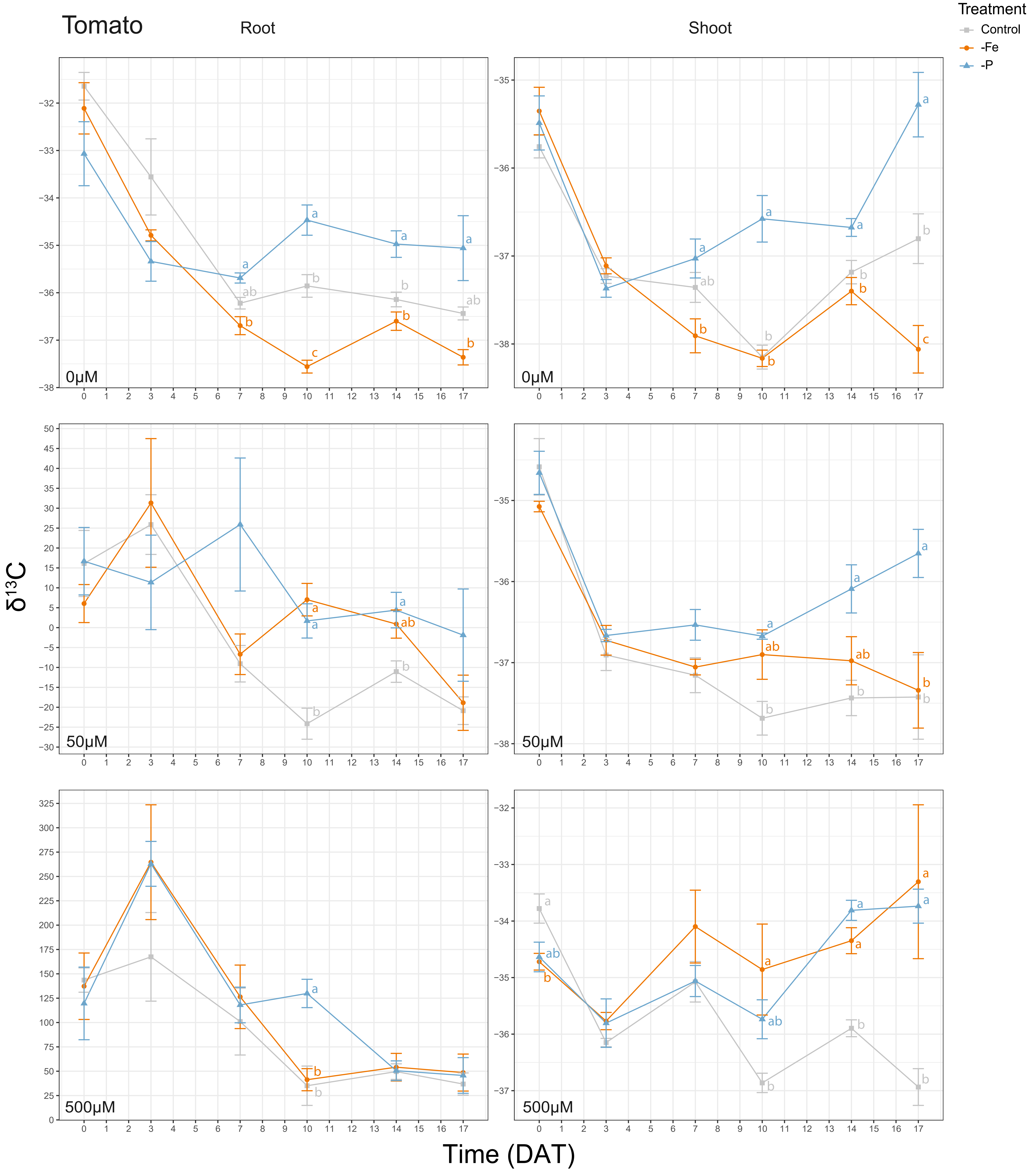

Figure S2: $\delta^{13}C$ values for tomato (*Solanum lycopersicon* L.) plants grown under control conditions (Control), phosphorus starvation (-P) and iron starvation (-Fe) plotted over time. Day 0 on the x-axis indicates the day on which some of the plants were transferred to the treatment-specific nutrient solutions (NSs), that is, after 7 days of growth in full NS. The $\delta^{13}C$ values of roots, left, and shoots, right, are represented as mean ± standard error, n = 7. The top two graphs refer to tomato plants exposed to ^{13}C Gly at 0 μmol L^{-1} (Millli-Q water), the middle two graphs to ^{13}C Gly at 50 μmol L^{-1}, while the bottom two to ^{13}C Gly at 500 μmol L^{-1} as reported in the bottom left of each graph. Letters next to the points indicate statistically significant differences ($p < 0.05$) between treatments assessed by a one-way ANOVA with Tukey post hoc test. DAT = Days After Treatment.
