# Supplementary material for: Exploring glycine root uptake dynamics in phosphorus and iron deficient tomato plants during the initial stages of plant development
Source: BMC Plant Biol. 2024 Jun 3;24:495. doi: 10.1186/s12870-024-05120-6 (PMC11145798; doi:10.1186/s12870-024-05120-6)
Supplement: Supplementary file 2 — Supplementary Material 2. [file 12870_2024_5120_MOESM2_ESM.pdf]

Table S1: Table summarizing the statistical analyses performed on the relative abundance of all elements detected with the Inductively Coupled Plasma (ICP) (Fig. 2). The table is divided in three sections: summary table for number of replicates (N), mean relative abundance, standard deviation (sd) and standard error (se), One-Way ANOVA on treatments, Tukey.HSD multiple comparison on treatments. C = Control condition; -P = phosphorus deficiency; -Fe = iron deficiency.

Summary statistics

Treatment	Tissue	Element	N	mean	sd	se
C	Root	Na	4	1658.05	198.19	99.10
C	Root	Mg	4	4629.50	181.60	90.80
C	Root	P	4	16204.00	581.11	290.56
C	Root	S	4	23039.50	379.29	189.65
C	Root	K	4	58792.50	2794.34	1397.17
C	Root	Ca	4	6641.25	222.18	111.09
C	Root	Ti	4	1.58	0.25	0.13
C	Root	Mn	4	221.95	32.05	16.03
C	Root	Fe	4	1527.73	210.71	105.36
C	Root	Cu	4	25.40	1.49	0.74
C	Root	Zn	4	160.73	24.24	12.12
C	Root	Se	4	1.23	0.98	0.49
C	Root	Mo	4	1.95	0.29	0.15
C	Shoot	Na	5	317.76	44.25	19.79
C	Shoot	Mg	5	5135.60	210.57	94.17
C	Shoot	P	5	6547.00	447.85	200.29
C	Shoot	S	5	9822.00	988.46	442.05
C	Shoot	K	5	54948.00	2778.59	1242.62
C	Shoot	Ca	5	33019.80	862.44	385.70
C	Shoot	Ti	5	0.57	0.09	0.04
C	Shoot	Mn	5	43.78	1.45	0.65
C	Shoot	Fe	5	162.66	12.21	5.46
C	Shoot	Cu	5	8.53	0.75	0.34
C	Shoot	Zn	5	36.58	5.79	2.59
C	Shoot	Se	5	0.72	0.61	0.27
C	Shoot	Mo	5	2.59	0.54	0.24
Fe	Root	Na	4	8308.76	3059.35	1529.68
Fe	Root	Mg	4	9485.75	837.97	418.99
Fe	Root	P	4	6214.00	883.02	441.51
Fe	Root	S	4	11911.50	1607.11	803.55
Fe	Root	K	4	50300.75	5976.65	2988.32
Fe	Root	Ca	4	13190.50	2432.44	1216.22
Fe	Root	Ti	4	2.92	1.34	0.67
Fe	Root	Mn	4	220.04	52.49	26.24
Fe	Root	Fe	4	388.76	101.06	50.53
Fe	Root	Cu	4	154.70	32.69	16.35
Fe	Root	Zn	4	1180.68	298.26	149.13
Fe	Root	Se	4	0.36	0.66	0.33
Fe	Root	Mo	4	3.23	0.68	0.34
Fe	Shoot	Na	5	1166.58	73.22	32.75

Fe	Shoot	Mg	5	6082.60	814.19	364.12
Fe	Shoot	P	5	6320.60	1060.84	474.42
Fe	Shoot	S	5	10593.80	633.60	283.35
Fe	Shoot	K	5	63270.00	13700.87	6127.22
Fe	Shoot	Ca	5	36123.40	5333.59	2385.25
Fe	Shoot	Ti	5	0.78	0.13	0.06
Fe	Shoot	Mn	5	82.12	27.16	12.15
Fe	Shoot	Fe	5	100.41	65.82	29.44
Fe	Shoot	Cu	5	12.92	2.42	1.08
Fe	Shoot	Zn	5	194.66	44.47	19.89
Fe	Shoot	Se	5	0.78	0.80	0.36
Fe	Shoot	Mo	5	3.94	0.51	0.23
P	Root	Na	4	9897.91	2432.67	1216.34
P	Root	Mg	4	13634.00	918.58	459.29
P	Root	P	4	1946.25	115.51	57.76
P	Root	S	4	10849.25	983.11	491.55
P	Root	K	4	46256.25	2619.83	1309.91
P	Root	Ca	4	13370.25	1666.45	833.22
P	Root	Ti	4	3.14	1.09	0.55
P	Root	Mn	4	98.84	18.07	9.04
P	Root	Fe	4	3860.21	281.76	140.88
P	Root	Cu	4	71.57	12.93	6.46
P	Root	Zn	4	862.77	142.22	71.11
P	Root	Se	4	2.00	1.70	0.85
P	Root	Mo	4	31.46	10.14	5.07
P	Shoot	Na	4	3874.00	886.34	443.17
P	Shoot	Mg	4	11222.00	638.26	319.13
P	Shoot	P	4	5004.75	270.60	135.30
P	Shoot	S	4	13900.00	594.08	297.04
P	Shoot	K	4	45373.25	656.67	328.33
P	Shoot	Ca	4	33579.75	1677.84	838.92
P	Shoot	Ti	4	1.06	0.14	0.07
P	Shoot	Mn	4	194.00	12.85	6.42
P	Shoot	Fe	4	648.39	60.60	30.30
P	Shoot	Cu	4	16.79	1.04	0.52
P	Shoot	Zn	4	156.97	53.72	26.86
P	Shoot	Se	4	0.87	0.19	0.10
P	Shoot	Mo	4	12.32	0.45	0.22

ICP_OneWayAnova_Results_Tr

Tissue	*Element*	*Statistical parameter*	*Treatment*	*Residuals*
**Root**	**Na**	*Df*	2	9
		F value	14.97	
		P value	0.001	
**Root**	**Mg**	*Df*	2	9
		F value	154.37	
		P value	0.000	
**Root**	**P**	*Df*	2	9
		F value	568.29	
		P value	0.000	
**Root**	**S**	*Df*	2	9
		F value	148.15	
		P value	0.000	
**Root**	**K**	*Df*	2	9
		F value	9.75	
		P value	0.006	
**Root**	**Ca**	*Df*	2	9
		F value	20.18	
		P value	0.000	
**Root**	**Ti**	*Df*	2	9
		F value	2.80	
		P value	0.113	
**Root**	**Mn**	*Df*	2	9
		F value	14.53	
		P value	0.002	
**Root**	**Fe**	*Df*	2	9
		F value	280.42	
		P value	0.000	
**Root**	**Cu**	*Df*	2	9
		F value	41.61	
		P value	0.000	
**Root**	**Zn**	*Df*	2	9
		F value	29.78	
		P value	0.000	
**Root**	**Se**	*Df*	2	9
		F value	1.88	
		P value	0.207	
**Root**	**Mo**	*Df*	2	9
		F value	32.31	
		P value	0.000	
**Shoot**	**Na**	*Df*	2	11
		F value	68.75	
		P value	0.000	
**Shoot**	**Mg**	*Df*	2	11
		F value	125.25	
		P value	0.000	
		Df	2	11

**Shoot**	**P**	*F value*	5.94	
		P value	0.018	
		Df	2	11
**Shoot**	**S**	*F value*	33.84	
		P value	0.000	
		Df	2	11
**Shoot**	**K**	*F value*	5.00	
		P value	0.029	
		Df	2	11
**Shoot**	**Ca**	*F value*	1.18	
		P value	0.343	
		Df	2	11
**Shoot**	**Ti**	*F value*	17.68	
		P value	0.000	
		Df	2	11
**Shoot**	**Mn**	*F value*	83.96	
		P value	0.000	
		Df	2	11
**Shoot**	**Fe**	*F value*	146.89	
		P value	0.000	
		Df	2	11
**Shoot**	**Cu**	*F value*	29.21	
		P value	0.000	
		Df	2	11
**Shoot**	**Zn**	*F value*	22.18	
		P value	0.000	
		Df	2	11
**Shoot**	**Se**	*F value*	0.06	
		P value	0.938	
		Df	2	11
**Shoot**	**Mo**	*F value*	468.10	
		P value	0.000	

Tukey.HSD multiple comparison on treatments

$Root

$Root$Na

	ppb	groups
P	9897.911	a
Fe	8308.762	a
C	1658.052	b

$Root$Mg

	ppb	groups
P	13634	a
Fe	9485.75	b
C	4629.5	c

$Root$P

	ppb	groups
C	16204	a
Fe	6214	b
P	1946.25	c

$Root$S

	ppb	groups
C	23039.5	a
Fe	11911.5	b
P	10849.25	b

$Root$K

	ppb	groups
C	58792.5	a
Fe	50300.75	b
P	46256.25	b

$Root$Ca

	ppb	groups
P	13370.25	a
Fe	13190.5	a
C	6641.25	b

$Root$Ti

	ppb	groups
P	3.14275	a
Fe	2.923	a
C	1.5835	a

$Root$Mn

	ppb	groups
C	221.9495	a
Fe	220.043	a
P	98.84425	b

$Root$Fe

	ppb	groups
P	3860.212	a
C	1527.7268	b
Fe	388.7635	c

$Root$Cu

	ppb	groups
Fe	154.70175	a
P	71.572	b
C	25.40025	c

$Root$Zn

	ppb	groups
Fe	1180.6827	a
P	862.7705	a
C	160.726	b

$Root$Se

	ppb	groups
P	1.9995	a
C	1.2285	a
Fe	0.3585	a

$Root$Mo

	ppb	groups
P	31.4615	a
Fe	3.23375	b
C	1.94725	b

$Shoot

$Shoot$Na

	ppb	groups
P	3873.9995	a
Fe	1166.5778	b
C	317.7584	c

$Shoot$Mg

	ppb	groups
P	11222	a
Fe	6082.6	b
C	5135.6	b

$Shoot$P

	ppb	groups
C	6547	a
Fe	6320.6	a
P	5004.75	b

$Shoot$S

	ppb	groups
P	13900	a
Fe	10593.8	b
C	9822	b

$Shoot$K

	ppb	groups
Fe	63270	a
C	54948	ab
P	45373.25	b

$Shoot$Ca

	ppb	groups
Fe	36123.4	a
P	33579.75	a
C	33019.8	a

$Shoot$Ti

	ppb	groups
P	1.059	a
Fe	0.7796	b
C	0.5716	b

$Shoot$Mn

	ppb	groups
P	193.9975	a
Fe	82.1214	b
C	43.7786	c

$Shoot$Fe

	ppb	groups
P	648.392	a
C	162.6566	b
Fe	100.4052	b

$Shoot$Cu

	ppb	groups
P	16.79025	a
Fe	12.9152	b
C	8.5258	c

$Shoot$Zn

	ppb	groups
Fe	194.6626	a
P	156.9702	a
C	36.583	b

$Shoot$Se

	ppb	groups
P	0.86825	a
Fe	0.782	a
C	0.721	a

$Shoot$Mo

	ppb	groups
P	12.32175	a
Fe	3.9394	b
C	2.5866	c
