# Supplementary material for: Exploring glycine root uptake dynamics in phosphorus and iron deficient tomato plants during the initial stages of plant development
Source: BMC Plant Biol. 2024 Jun 3;24:495. doi: 10.1186/s12870-024-05120-6 (PMC11145798; doi:10.1186/s12870-024-05120-6)
Supplement: Supplementary file 3 — Supplementary Material 3. [file 12870_2024_5120_MOESM3_ESM.pdf]

***Table S2:*** Table summarizing the statistical analyses performed on the results obtained by the Infra-Red Gas Analyzer (IRGA), namely photosynthetic rate (A), transpiration rate (E) and stomatal conductance (gs) (Fig. 3). The table is divided in three sections: summary table for number of replicates (N), mean relative abundance, standard deviation (sd) and standard error (se), One-Way ANOVA on treatments, Tukey.HSD multiple comparison on treatments. C = Control condition; -P = phosphorus deficiency; -Fe = iron deficiency.

***Summary table***

| Treatment | Variable | N | mean | sd | se |
|---|---|---|---|---|---|
| C | E | 9 | 0.394 | 0.169 | 0.056 |
| C | A | 9 | 2.073 | 0.459 | 0.153 |
| C | gs | 9 | 0.018 | 0.008 | 0.003 |
| Fe | E | 11 | 0.415 | 0.187 | 0.056 |
| Fe | A | 11 | 1.364 | 0.188 | 0.057 |
| Fe | gs | 11 | 0.018 | 0.008 | 0.002 |
| P | E | 9 | 0.196 | 0.138 | 0.046 |
| P | A | 10 | 1.035 | 0.584 | 0.185 |
| P | gs | 9 | 0.009 | 0.006 | 0.002 |

**Physiological_Parameters_OneWay_ANOVA**

| *Variable* | *atistical parame* | *Treatment* | *Residuals* |
|---|---|---|---|
| **E** | *Df* | 2 | 27 |
| | *F value* | 6.37 | |
| | *P value* | 0.005 | |
| **A** | *Df* | 2 | 27 |
| | *F value* | 14.00 | |
| | *P value* | 0.000 | |
| **gs** | *Df* | 2 | 27 |
| | *F value* | 6.40 | |
| | *P value* | 0.005 | |

***Tukey.HSDmultiplecomparison***

***$E***

| | value | groups |
|---|---|---|
| Fe | 0.4148496 | a |
| C | 0.394206 | a |
| P | 0.169402 | b |

***$A***

| | value | groups |
|---|---|---|
| C | 2.073333 | a |
| Fe | 1.363636 | b |
| P | 1.035 | b |

***$gs***

| | value | groups |
|---|---|---|
| Fe | 0.018262985 | a |
| C | 0.017671688 | a |
| P | 0.007464612 | b |
