# Supplementary material for: Exploring glycine root uptake dynamics in phosphorus and iron deficient tomato plants during the initial stages of plant development
Source: BMC Plant Biol. 2024 Jun 3;24:495. doi: 10.1186/s12870-024-05120-6 (PMC11145798; doi:10.1186/s12870-024-05120-6)
Supplement: Supplementary file 4 — Supplementary Material 4. [file 12870_2024_5120_MOESM4_ESM.pdf]

***Table S3:*** Table summarizing the statistical analyses performed on the Mass Balance (Fig. 4). The table is divided in six sections: summary table for number of replicates (N), mean mass balance, standard deviation (sd) and standard error (se), Two-Way ANOVA, One-Way ANOVA on treatments, Tukey.HSD multiple comparison on treatments, One-Way ANOVA on time (plant development) and Tukey.HSD multiple comparison on time (plant development). C = Control condition; -P = phosphorus deficiency; -Fe = iron deficiency.

***Summary table***

| Treatment | Time | Glycine concentration (µM) | Tissue | N | Mass balance (mg $g^{-1}$ Gly derived 13C $DW^{-1}$) | sd | se |
|---|---|---|---|---|---|---|---|
| C | 0 | 500 | Root | 5 | 1.0429 | 0.2125 | 0.0950 |
| C | 0 | 500 | Shoot | 5 | 0.0107 | 0.0032 | 0.0014 |
| C | 0 | 50 | Root | 5 | 0.2693 | 0.1072 | 0.0479 |
| C | 0 | 50 | Shoot | 5 | 0.0066 | 0.0044 | 0.0020 |
| C | 3 | 500 | Root | 5 | 0.9307 | 0.4244 | 0.1898 |
| C | 3 | 500 | Shoot | 5 | 0.0058 | 0.0010 | 0.0004 |
| C | 3 | 50 | Root | 5 | 0.2802 | 0.0563 | 0.0252 |
| C | 3 | 50 | Shoot | 5 | 0.0017 | 0.0023 | 0.0010 |
| C | 7 | 500 | Root | 5 | 0.7341 | 0.4042 | 0.1808 |
| C | 7 | 500 | Shoot | 5 | 0.0116 | 0.0040 | 0.0018 |
| C | 7 | 50 | Root | 5 | 0.1580 | 0.0589 | 0.0264 |
| C | 7 | 50 | Shoot | 5 | 0.0010 | 0.0024 | 0.0011 |
| C | 10 | 500 | Root | 5 | 0.4086 | 0.2241 | 0.1002 |
| C | 10 | 500 | Shoot | 5 | 0.0066 | 0.0021 | 0.0009 |
| C | 10 | 50 | Root | 5 | 0.0724 | 0.0519 | 0.0232 |
| C | 10 | 50 | Shoot | 5 | 0.0024 | 0.0024 | 0.0011 |
| C | 14 | 500 | Root | 5 | 0.4718 | 0.0851 | 0.0381 |
| C | 14 | 500 | Shoot | 5 | 0.0066 | 0.0016 | 0.0007 |
| C | 14 | 50 | Root | 5 | 0.1382 | 0.0324 | 0.0145 |
| C | 14 | 50 | Shoot | 5 | -0.0013 | 0.0025 | 0.0011 |
| C | 17 | 500 | Root | 5 | 0.4276 | 0.1342 | 0.0600 |
| C | 17 | 500 | Shoot | 5 | -0.0009 | 0.0040 | 0.0018 |
| C | 17 | 50 | Root | 5 | 0.0884 | 0.0386 | 0.0173 |
| C | 17 | 50 | Shoot | 5 | -0.0038 | 0.0058 | 0.0026 |
| Fe | 0 | 500 | Root | 5 | 0.8591 | 0.3774 | 0.1688 |
| Fe | 0 | 500 | Shoot | 5 | 0.0033 | 0.0017 | 0.0008 |
| Fe | 0 | 50 | Root | 5 | 0.1982 | 0.0473 | 0.0212 |
| Fe | 0 | 50 | Shoot | 5 | 0.0016 | 0.0008 | 0.0004 |
| Fe | 3 | 500 | Root | 5 | 1.3434 | 0.6563 | 0.2935 |
| Fe | 3 | 500 | Shoot | 5 | 0.0072 | 0.0018 | 0.0008 |
| Fe | 3 | 50 | Root | 5 | 0.3417 | 0.1613 | 0.0721 |
| Fe | 3 | 50 | Shoot | 5 | 0.0021 | 0.0021 | 0.0009 |
| Fe | 7 | 500 | Root | 5 | 0.9140 | 0.4054 | 0.1813 |
| Fe | 7 | 500 | Shoot | 5 | 0.0199 | 0.0071 | 0.0032 |
| Fe | 7 | 50 | Root | 5 | 0.1768 | 0.0653 | 0.0292 |
| Fe | 7 | 50 | Shoot | 5 | 0.0044 | 0.0012 | 0.0005 |
| Fe | 10 | 500 | Root | 5 | 0.4618 | 0.1379 | 0.0617 |
| Fe | 10 | 500 | Shoot | 5 | 0.0167 | 0.0087 | 0.0039 |

| | | | | | | | |
|---|---|---|---|---|---|---|---|
| Fe | 10 | 50 | Root | 5 | 0.2500 | 0.0364 | 0.0163 |
| Fe | 10 | 50 | Shoot | 5 | 0.0061 | 0.0032 | 0.0014 |
| Fe | 14 | 500 | Root | 5 | 0.5331 | 0.1890 | 0.0845 |
| Fe | 14 | 500 | Shoot | 5 | 0.0156 | 0.0027 | 0.0012 |
| Fe | 14 | 50 | Root | 5 | 0.2256 | 0.0410 | 0.0183 |
| Fe | 14 | 50 | Shoot | 5 | 0.0020 | 0.0032 | 0.0014 |
| Fe | 17 | 500 | Root | 4 | 0.4971 | 0.2045 | 0.1023 |
| Fe | 17 | 500 | Shoot | 5 | 0.0223 | 0.0125 | 0.0056 |
| Fe | 17 | 50 | Root | 4 | 0.1088 | 0.0834 | 0.0417 |
| Fe | 17 | 50 | Shoot | 5 | 0.0031 | 0.0052 | 0.0023 |
| P | 0 | 500 | Root | 5 | 0.8506 | 0.4995 | 0.2234 |
| P | 0 | 500 | Shoot | 5 | 0.0045 | 0.0032 | 0.0014 |
| P | 0 | 50 | Root | 5 | 0.2599 | 0.0953 | 0.0426 |
| P | 0 | 50 | Shoot | 5 | 0.0047 | 0.0033 | 0.0015 |
| P | 3 | 500 | Root | 5 | 1.4708 | 0.3001 | 0.1342 |
| P | 3 | 500 | Shoot | 5 | 0.0091 | 0.0064 | 0.0029 |
| P | 3 | 50 | Root | 5 | 0.2501 | 0.1461 | 0.0653 |
| P | 3 | 50 | Shoot | 5 | 0.0037 | 0.0007 | 0.0003 |
| P | 7 | 500 | Root | 5 | 0.8634 | 0.2106 | 0.0942 |
| P | 7 | 500 | Shoot | 5 | 0.0109 | 0.0034 | 0.0015 |
| P | 7 | 50 | Root | 4 | 0.3546 | 0.1923 | 0.0962 |
| P | 7 | 50 | Shoot | 5 | 0.0027 | 0.0023 | 0.0010 |
| P | 10 | 500 | Root | 5 | 0.8654 | 0.2043 | 0.0914 |
| P | 10 | 500 | Shoot | 5 | 0.0047 | 0.0043 | 0.0019 |
| P | 10 | 50 | Root | 5 | 0.1969 | 0.0507 | 0.0227 |
| P | 10 | 50 | Shoot | 5 | -0.0005 | 0.0005 | 0.0002 |
| P | 14 | 500 | Root | 5 | 0.4860 | 0.1281 | 0.0573 |
| P | 14 | 500 | Shoot | 5 | 0.0155 | 0.0019 | 0.0008 |
| P | 14 | 50 | Root | 5 | 0.2111 | 0.0560 | 0.0250 |
| P | 14 | 50 | Shoot | 5 | 0.0031 | 0.0035 | 0.0016 |
| P | 17 | 500 | Root | 5 | 0.4451 | 0.2106 | 0.0942 |
| P | 17 | 500 | Shoot | 5 | 0.0076 | 0.0029 | 0.0013 |
| P | 17 | 50 | Root | 5 | 0.1828 | 0.1380 | 0.0617 |
| P | 17 | 50 | Shoot | 5 | -0.0020 | 0.0036 | 0.0016 |

**Two-Way ANOVA**

| *Tissue* | *Gly Concentration (μM)* | *Statistical parameter* | *Treatment* | *Time* | *Treatment:Time* | *Residuals* |
|---|---|---|---|---|---|---|
| **Root** | **50** | *Df* | 2 | 5 | 10 | 70 |
| | | *F value* | 4.79 | 5.56 | 2.23 | |
| | | *P value* | 0.011 | 0.000 | 0.025 | |
| **Root** | **500** | *Df* | 2 | 5 | 10 | 72 |
| | | *F value* | 3.53 | 4.83 | 3.46 | |
| | | *P value* | 0.034 | 0.001 | 0.001 | |
| **Shoot** | **50** | *Df* | 2 | 5 | 10 | 71 |
| | | *F value* | 2.02 | 13.75 | 1.25 | |
| | | *P value* | 0.141 | 0.000 | 0.274 | |
| **Shoot** | **500** | *Df* | 2 | 5 | 10 | 72 |
| | | *F value* | 18.28 | 5.60 | 6.55 | |
| | | *P value* | 0.000 | 0.000 | 0.000 | |

**One-Way ANOVA Treatment**

| *Tissue* | *Gly Concentration (µM)* | *Time* | *Statistical parameter* | *Treatment* | *Residuals* |
|---|---|---|---|---|---|
| **Root** | **50** | **0** | *Df* | 2 | 12 |
| | | | *F value* | 0.98 | |
| | | | *P value* | 0.403 | |
| **Root** | **50** | **3** | *Df* | 2 | 12 |
| | | | *F value* | 0.65 | |
| | | | *P value* | 0.541 | |
| **Root** | **50** | **7** | *Df* | 2 | 12 |
| | | | *F value* | 3.91 | |
| | | | *P value* | 0.052 | |
| **Root** | **50** | **10** | *Df* | 2 | 12 |
| | | | *F value* | 18.92 | |
| | | | *P value* | 0.000 | |
| **Root** | **50** | **14** | *Df* | 2 | 12 |
| | | | *F value* | 5.62 | |
| | | | *P value* | 0.019 | |
| **Root** | **50** | **17** | *Df* | 2 | 12 |
| | | | *F value* | 1.30 | |
| | | | *P value* | 0.312 | |
| **Root** | **500** | **0** | *Df* | 2 | 12 |
| | | | *F value* | 0.41 | |
| | | | *P value* | 0.676 | |
| **Root** | **500** | **3** | *Df* | 2 | 12 |
| | | | *F value* | 1.71 | |
| | | | *P value* | 0.223 | |
| **Root** | **500** | **7** | *Df* | 2 | 12 |
| | | | *F value* | 0.35 | |
| | | | *P value* | 0.714 | |
| **Root** | **500** | **10** | *Df* | 2 | 12 |
| | | | *F value* | 8.43 | |
| | | | *P value* | 0.005 | |
| **Root** | **500** | **14** | *Df* | 2 | 12 |
| | | | *F value* | 0.26 | |
| | | | *P value* | 0.776 | |
| **Root** | **500** | **17** | *Df* | 2 | 12 |
| | | | *F value* | 0.17 | |
| | | | *P value* | 0.849 | |
| **Shoot** | **50** | **0** | *Df* | 2 | 12 |
| | | | *F value* | 3.13 | |
| | | | *P value* | 0.081 | |
| **Shoot** | **50** | **3** | *Df* | 2 | 12 |
| | | | *F value* | 1.63 | |
| | | | *P value* | 0.237 | |
| **Shoot** | **50** | **7** | *Df* | 2 | 12 |
| | | | *F value* | 3.44 | |
| | | | *P value* | 0.066 | |

| | | | | | |
|---|---|---|---|---|---|
| **Shoot** | **50** | **10** | *Df* | 2 | 12 |
| | | | *F value* | 10.25 | |
| | | | *P value* | 0.003 | |
| **Shoot** | **50** | **14** | *Df* | 2 | 12 |
| | | | *F value* | 2.76 | |
| | | | *P value* | 0.103 | |
| **Shoot** | **50** | **17** | *Df* | 2 | 12 |
| | | | *F value* | 2.67 | |
| | | | *P value* | 0.110 | |
| **Shoot** | **500** | **0** | *Df* | 2 | 12 |
| | | | *F value* | 10.04 | |
| | | | *P value* | 0.003 | |
| **Shoot** | **500** | **3** | *Df* | 2 | 12 |
| | | | *F value* | 0.90 | |
| | | | *P value* | 0.431 | |
| **Shoot** | **500** | **7** | *Df* | 2 | 12 |
| | | | *F value* | 4.82 | |
| | | | *P value* | 0.029 | |
| **Shoot** | **500** | **10** | *Df* | 2 | 12 |
| | | | *F value* | 6.39 | |
| | | | *P value* | 0.013 | |
| **Shoot** | **500** | **14** | *Df* | 2 | 12 |
| | | | *F value* | 30.75 | |
| | | | *P value* | 0.000 | |
| **Shoot** | **500** | **17** | *Df* | 2 | 12 |
| | | | *F value* | 11.36 | |
| | | | *P value* | 0.002 | |

***Tukey.HSD multiple comparison on treatments***

***$`50µM_TR`***

*$`50µM_TR`$`0`*

| | Value | groups |
|---|---|---|
| C | 0.269343 | a |
| P | 0.259927 | a |
| Fe | 0.198181 | a |

*$`50µM_TR`$`3`*

| | Value | groups |
|---|---|---|
| Fe | 0.341673 | a |
| C | 0.280206 | a |
| P | 0.250103 | a |

*$`50µM_TR`$`7`*

| | Value | groups |
|---|---|---|
| P | 0.354568 | a |
| Fe | 0.176758 | a |
| C | 0.157975 | a |

*$`50µM_TR`$`10`*

| | Value | groups |
|---|---|---|
| Fe | 0.249972 | a |
| P | 0.196916 | a |
| C | 0.07237 | b |

*$`50µM_TR`$`14`*

| | Value | groups |
|---|---|---|
| Fe | 0.225647 | a |
| P | 0.211072 | ab |
| C | 0.138188 | b |

*$`50µM_TR`$`17`*

| | Value | groups |
|---|---|---|
| P | 0.182799 | a |
| Fe | 0.108835 | a |
| C | 0.088446 | a |

***$`50µM_TS`***

*$`50µM_TS`$`0`*

| | Value | groups |
|---|---|---|
| C | 0.006631 | a |
| P | 0.004742 | a |
| Fe | 0.001604 | a |

*$`50µM_TS`$`3`*

| | Value | groups |
|---|---|---|
| P | 0.003697 | a |

| | | |
|---|---|---|
| Fe | 0.002069 | a |
| C | 0.001725 | a |

*$`50µM_TS`$`7`*

| | Value | groups |
|---|---|---|
| Fe | 0.004355 | a |
| P | 0.002717 | a |
| C | 0.00095 | a |

*$`50µM_TS`$`10`*

| | Value | groups |
|---|---|---|
| Fe | 0.006084 | a |
| C | 0.0024 | ab |
| P | -0.00054 | b |

*$`50µM_TS`$`14`*

| | Value | groups |
|---|---|---|
| P | 0.003141 | a |
| Fe | 0.002002 | a |
| C | -0.00126 | a |

*$`50µM_TS`$`17`*

| | Value | groups |
|---|---|---|
| Fe | 0.003149 | a |
| P | -0.002 | a |
| C | -0.00384 | a |

***$`500µM_TR`***

*$`500µM_TR`$`0`*

| | Value | groups |
|---|---|---|
| C | 1.042941 | a |
| Fe | 0.859122 | a |
| P | 0.850604 | a |

*$`500µM_TR`$`3`*

| | Value | groups |
|---|---|---|
| P | 1.470801 | a |
| Fe | 1.343419 | a |
| C | 0.930735 | a |

*$`500µM_TR`$`7`*

| | Value | groups |
|---|---|---|
| Fe | 0.913961 | a |
| P | 0.863419 | a |
| C | 0.734077 | a |

*$`500µM_TR`$`10`*

| | Value | groups |
|---|---|---|
| P | 0.865389 | a |

| | | |
|---|---|---|
| Fe | 0.461816 | b |
| C | 0.408597 | b |

*$`500µM_TR`$`14`*

| | Value | groups |
|---|---|---|
| Fe | 0.533093 | a |
| P | 0.486007 | a |
| C | 0.471831 | a |

*$`500µM_TR`$`17`*

| | Value | groups |
|---|---|---|
| Fe | 0.497144 | a |
| P | 0.445067 | a |
| C | 0.427553 | a |

***$`500µM_TS`***

*$`500µM_TS`$`0`*

| | Value | groups |
|---|---|---|
| C | 0.010697 | a |
| P | 0.004538 | b |
| Fe | 0.003334 | b |

*$`500µM_TS`$`3`*

| | Value | groups |
|---|---|---|
| P | 0.009098 | a |
| Fe | 0.0072 | a |
| C | 0.005786 | a |

*$`500µM_TS`$`7`*

| | Value | groups |
|---|---|---|
| Fe | 0.019886 | a |
| C | 0.011551 | ab |
| P | 0.010901 | b |

*$`500µM_TS`$`10`*

| | Value | groups |
|---|---|---|
| Fe | 0.016745 | a |
| C | 0.006584 | b |
| P | 0.004738 | b |

*$`500µM_TS`$`14`*

| | Value | groups |
|---|---|---|
| Fe | 0.015648 | a |
| P | 0.015533 | a |
| C | 0.006598 | b |

*$`500µM_TS`$`17`*

| | Value | groups |
|---|---|---|
| Fe | 0.022254 | a |

| | | |
|---|---|---|
| P | 0.00762 | b |
| C | -0.0009 | b |

**One-Way ANOVA Time**

| *Tissue* | *Gly Concentration (µM)* | *Treatment* | *Statistical parameter* | *Time* | *Residuals* |
|---|---|---|---|---|---|
| **Root** | **50** | **C** | *Df* | 5 | 24 |
| | | | *F value* | 10.10 | |
| | | | *P value* | 0.000 | |
| **Root** | **50** | **Fe** | *Df* | 5 | 23 |
| | | | *F value* | 3.95 | |
| | | | *P value* | 0.010 | |
| **Root** | **50** | **P** | *Df* | 5 | 23 |
| | | | *F value* | 1.18 | |
| | | | *P value* | 0.351 | |
| **Root** | **500** | **C** | *Df* | 5 | 24 |
| | | | *F value* | 4.88 | |
| | | | *P value* | 0.003 | |
| **Root** | **500** | **Fe** | *Df* | 5 | 23 |
| | | | *F value* | 3.96 | |
| | | | *P value* | 0.010 | |
| **Root** | **500** | **P** | *Df* | 5 | 24 |
| | | | *F value* | 8.39 | |
| | | | *P value* | 0.000 | |
| **Shoot** | **50** | **C** | *Df* | 5 | 24 |
| | | | *F value* | 4.92 | |
| | | | *P value* | 0.003 | |
| **Shoot** | **50** | **Fe** | *Df* | 5 | 24 |
| | | | *F value* | 1.66 | |
| | | | *P value* | 0.182 | |
| **Shoot** | **50** | **P** | *Df* | 5 | 24 |
| | | | *F value* | 4.99 | |
| | | | *P value* | 0.003 | |
| **Shoot** | **500** | **C** | *Df* | 5 | 24 |
| | | | *F value* | 11.71 | |
| | | | *P value* | 0.000 | |
| **Shoot** | **500** | **Fe** | *Df* | 5 | 24 |
| | | | *F value* | 5.53 | |
| | | | *P value* | 0.002 | |
| **Shoot** | **500** | **P** | *Df* | 5 | 24 |
| | | | *F value* | 5.53 | |
| | | | *P value* | 0.002 | |

***Tukey.HSD multiple comparison on time***

***$`50µM_TR`***

*$`50µM_TR`$C*

| | Value | groups |
|---|---|---|
| 3 | 0.280206 | a |
| 0 | 0.269343 | ab |
| 7 | 0.157975 | bc |
| 14 | 0.138188 | c |
| 17 | 0.088446 | c |
| 10 | 0.07237 | c |

*$`50µM_TR`$Fe*

| | Value | groups |
|---|---|---|
| 3 | 0.341673 | a |
| 10 | 0.249972 | ab |
| 14 | 0.225647 | ab |
| 0 | 0.198181 | ab |
| 7 | 0.176758 | ab |
| 17 | 0.108835 | b |

*$`50µM_TR`$P*

| | Value | groups |
|---|---|---|
| 7 | 0.354568 | a |
| 0 | 0.259927 | a |
| 3 | 0.250103 | a |
| 14 | 0.211072 | a |
| 10 | 0.196916 | a |
| 17 | 0.182799 | a |

***$`50µM_TS`***

*$`50µM_TS`$C*

| | Value | groups |
|---|---|---|
| 0 | 0.006631 | a |
| 10 | 0.0024 | ab |
| 3 | 0.001725 | ab |
| 7 | 0.00095 | ab |
| 14 | -0.00126 | b |
| 17 | -0.00384 | b |

*$`50µM_TS`$Fe*

| | Value | groups |
|---|---|---|
| 10 | 0.006084 | a |
| 7 | 0.004355 | a |
| 17 | 0.003149 | a |
| 3 | 0.002069 | a |
| 14 | 0.002002 | a |
| 0 | 0.001604 | a |

*$`50µM_TS`$P*

| | Value | groups |
|---|---|---|
| 0 | 0.004742 | a |
| 3 | 0.003697 | ab |
| 14 | 0.003141 | abc |
| 7 | 0.002717 | abc |
| 10 | -0.00054 | bc |
| 17 | -0.002 | c |

***$`500µM_TR`***

*$`500µM_TR`$C*

| | Value | groups |
|---|---|---|
| 0 | 1.042941 | a |
| 3 | 0.930735 | ab |
| 7 | 0.734077 | ab |
| 14 | 0.471831 | b |
| 17 | 0.427553 | b |
| 10 | 0.408597 | b |

*$`500µM_TR`$Fe*

| | Value | groups |
|---|---|---|
| 3 | 1.343419 | a |
| 7 | 0.913961 | ab |
| 0 | 0.859122 | ab |
| 14 | 0.533093 | b |
| 17 | 0.497144 | b |
| 10 | 0.461816 | b |

*$`500µM_TR`$P*

| | Value | groups |
|---|---|---|
| 3 | 1.470801 | a |
| 10 | 0.865389 | b |
| 7 | 0.863419 | b |
| 0 | 0.850604 | b |
| 14 | 0.486007 | b |
| 17 | 0.445067 | b |

***$`500µM_TS`***

*$`500µM_TS`$C*

| | Value | groups |
|---|---|---|
| 7 | 0.011551 | a |
| 0 | 0.010697 | ab |
| 14 | 0.006598 | ab |
| 10 | 0.006584 | ab |
| 3 | 0.005786 | b |
| 17 | -0.0009 | c |

*$`500µM_TS`$Fe*

| | Value | groups |
|---|---|---|
| 17 | 0.022254 | a |
| 7 | 0.019886 | ab |
| 10 | 0.016745 | abc |
| 14 | 0.015648 | abc |
| 3 | 0.0072 | bc |
| 0 | 0.003334 | c |

*$`500µM_TS`$P*

| | Value | groups |
|---|---|---|
| 14 | 0.015533 | a |
| 7 | 0.010901 | ab |
| 3 | 0.009098 | ab |
| 17 | 0.00762 | b |
| 10 | 0.004738 | b |
| 0 | 0.004538 | b |
