# Supplementary material for: Exploring glycine root uptake dynamics in phosphorus and iron deficient tomato plants during the initial stages of plant development
Source: BMC Plant Biol. 2024 Jun 3;24:495. doi: 10.1186/s12870-024-05120-6 (PMC11145798; doi:10.1186/s12870-024-05120-6)
Supplement: Supplementary file 5 — Supplementary Material 5. [file 12870_2024_5120_MOESM5_ESM.pdf]

Table S4: Table summarizing the statistical analyses performed on the regression models run with Mass Balance data (Fig. 4). The table is divided in two sections: One-Way ANCOVA on treatment and Tukey.HSD multiple comparison on treatments slope. C = Control condition; -P = phosphorus deficiency; -Fe = iron deficiency.

One-Way ANCOVA on treatment

TR50microM
$C

Call:
lm(formula = Value ~ Time, data = i)

Residuals:

	Min	1Q	Median	3Q	Max
	-0.16193	-0.0436	0.00116	0.05711	0.14742

Coefficients:

	Estimate	Std. Error	t value	Pr(>|t|)	
(Intercept)	0.26704	0.02319	11.515	3.92E-12	***
Time	-0.01168	0.00224	-5.214	1.54E-05	***

Signif. codes: 0 ‘***’ 0.001 ‘**’ 0.01 ‘*’ 0.05 ‘.’ 0.1 ‘’ 1

Residual standard error:	0.0725	on 28 degrees of freedom
MultipleR-squared:	0.4927	
Adjusted R-squared:	0.4745	
F-statistic:	27.19	on 1 and 28 DF
p-value:	1.54E-05	

$P

Call:
lm(formula = Value ~ Time, data = i)

Residuals:

	Min	1Q	Median	3Q	Max
	-0.18498	-0.0669	-0.0266	0.04214	0.3761

Coefficients:

	Estimate	Std. Error	t value	Pr(>|t|)	
(Intercept)	0.282557	0.03879	7.284	7.80E-08	***
Time	-0.005128	0.00371	-1.381	0.179	

Signif. codes: 0 ‘***’ 0.001 ‘**’ 0.01 ‘*’ 0.05 ‘.’ 0.1 ‘’ 1

Residual standard error:	0.12	on 27 degrees of freedom

MultipleR-squared:	0.066	
Adjusted R-squared:	0.03141	
F-statistic:	1.908	on 1 and 27 DF
p-value:	0.1785	

$Fe

Call:
lm(formula = Value ~ Time, data = i)

Residuals:

	Min		1Q	Median	3Q	Max
		-0.148465	-0.073	-0.0004	0.04959	0.23594

Coefficients:

	Estimate Std. Error		t	value	Pr(>|t|)	
(Intercept)		0.266878	0.03231	8.259	7.25E-09	***
Time		-0.005643	0.00322	-1.754	0.0908	.

Signif.	codes:		0	'***'	0.001	'**'	0.01	'*'
			0.05	'.'	0.1	''	1	

Residual standard error:	0.1003	on 27 degrees of freedom
MultipleR-squared:	0.1023	
Adjusted R-squared:	0.06904	
F-statistic:	3.077	on 1 and 27 DF
p-value:	0.09077	

TS50microM
$C

Call:
lm(formula = Value ~ Time, data = i)

Residuals:

	Min		1Q	Median	3Q	Max
		-0.005522	-0.0027	0.00017	0.00175	0.00871

Coefficients:

	Estimate Std. Error		t	value	Pr(>|t|)	
(Intercept)		0.0052891	0.00115	4.583	8.66E-05	***
Time		-0.0004927	0.00011	-4.419	0.00014	***

Signif.	codes:		0	'***'	0.001	'**'	0.01	'*'
			0.05	'.'	0.1	''	1	

Residual standard error:	0.003608	on 28 degrees of freedom
MultipleR-squared:	0.4109	

Adjusted R-squared:		0.3899					
F-statistic:		19.53	on 1 and 28 DF				
p-value:		0.0001353					

$P

Call:
lm(formula = Value ~ Time, data = i)

Residuals:

	Min		1Q	Median	3Q	Max	
		-0.0051411	-0.002	-6E-05	0.001	0.00888	

Coefficients:

	Estimate Std. Error		t	value	Pr(>|t|)		
(Intercept)		4.61E-03	9.33E-04	4.938	3.28E-05	***	
Time		-3.12E-04	9.01E-05	-3.456	0.00177	**	

Signif.	codes:		0	‘***’	0.001	‘**’	0.01 ‘*’
			0.05	‘.’	0.1	‘’	1

Residual standard error:		0.002917	on 28 degrees of freedom
MultipleR-squared:		2.99E-01	
Adjusted R-squared:		0.274	
F-statistic:		1.20E+01	on 1 and 28 DF
p-value:		0.001765	

$Fe

Call:
lm(formula = Value ~ Time, data = i)

Residuals:

	Min		1Q	Median	3Q	Max	
		-0.0091541	-0.0016	1.8E-05	0.00184	0.00661	

Coefficients:

	Estimate Std. Error		t	value	Pr(>|t|)		
(Intercept)		2.59E-03	1.02E-03	2.538	0.017	*	
Time		7.33E-05	9.85E-05	0.745	0.463		

Signif.	codes:		0	‘***’	0.001	‘**’	0.01 ‘*’
			0.05	‘.’	0.1	‘’	1

Residual standard error:		0.003187	on 28 degrees of freedom
MultipleR-squared:		1.94E-02	
Adjusted R-squared:		-0.01559	
F-statistic:		5.55E-01	on 1 and 28 DF

p-value:	0.4626

TR500microM
$C

Call:
lm(formula = Value ~ Time, data = i)

Residuals:

	Min	1Q	Median	3Q	Max
	-0.52737	-0.2354	-0.0031	0.17936	0.59682

Coefficients:

	Estimate	Std. Error	t value	Pr(>|t|)	
(Intercept)	1.00373	0.08806	11.398	4.97E-12	***
Time	-0.039346	0.00851	-4.625	7.72E-05	***

Signif.	codes:	0	'***'	0.001	'**'	0.01	'*'
		0.05	'.'	0.1	''	1	

Residual standard error:	0.2753	on 28 degrees of freedom
MultipleR-squared:	0.4331	
Adjusted R-squared:	0.4129	
F-statistic:	21.39	on 1 and 28 DF
p-value:	7.72E-05	

$P

Call:
lm(formula = Value ~ Time, data = i)

Residuals:

	Min	1Q	Median	3Q	Max
	-0.85017	-0.1318	-0.0331	0.17305	0.58831

Coefficients:

	Estimate	Std. Error	t value	Pr(>|t|)	
(Intercept)	1.18971	0.11263	10.563	2.83E-11	***
Time	-0.04229	0.01088	-3.887	0.00057	***

Signif.	codes:	0	'***'	0.001	'**'	0.01	'*'
		0.05	'.'	0.1	''	1	

Residual standard error:	0.3521	on 28 degrees of freedom
MultipleR-squared:	0.3505	
Adjusted R-squared:	0.3273	
F-statistic:	15.11	on 1 and 28 DF
p-value:	0.0005679	

$Fe

Call:
lm(formula = Value ~ Time, data = i)

Residuals:

	Min	1Q	Median	3Q	Max
	-0.61749	-0.2791	-0.0807	0.28243	0.97925

Coefficients:

	Estimate Std. Error	t	value	Pr(>|t|)	
(Intercept)	1.10374	0.13287	8.307	6.47E-09	***
Time	-0.03976	0.01323	-3.006	0.00567	**

Signif.	codes:	0	‘***’	0.001	‘**’	0.01	‘*’
		0.05	‘.’	0.1	‘’	1	

Residual standard error:	0.4126	on 27 degrees of freedom
MultipleR-squared:	0.2507	
Adjusted R-squared:	0.223	
F-statistic:	9.034	on 1 and 27 DF
p-value:	0.005666	

TS500microM
$C

Call:
lm(formula = Value ~ Time, data = i)

Residuals:

	Min	1Q	Median	3Q	Max
	-0.008297	-0.0027	0.00026	0.00254	0.00774

Coefficients:

	Estimate Std. Error	t	value	Pr(>|t|)	
(Intercept)	0.0108401	0.00128	8.494	3.10E-09	***
Time	-0.0004848	0.00012	-3.933	0.0005	***

Signif.	codes:	0	‘***’	0.001	‘**’	0.01	‘*’
		0.05	‘.’	0.1	‘’	1	

Residual standard error:	0.00399	on 28 degrees of freedom
MultipleR-squared:	0.3558	
Adjusted R-squared:	0.3328	
F-statistic:	15.47	on 1 and 28 DF
p-value:	0.0005029	

$P

Call:
lm(formula = Value ~ Time, data = i)

Residuals:

Min	1Q	Median	3Q	Max
-0.009179	-0.004	-0.0008	0.00359	0.0132

Coefficients:

	Estimate	Std. Error	t value	Pr(>|t|)	
(Intercept)	0.0066145	0.00164	4.032	0.00039	***
Time	0.0002498	0.00016	1.577	0.12613	

Signif.	codes:	0	'***'	0.001	'**'	0.01	'*'
		0.05	'.'	0.1	''	1	

Residual standard error:	0.005129	on 28 degrees of freedom
MultipleR-squared:	0.08153	
Adjusted R-squared:	0.04873	
F-statistic:	2.486	on 1 and 28 DF
p-value:	0.1261	

$Fe

Call:
lm(formula = Value ~ Time, data = i)

Residuals:

Min	1Q	Median	3Q	Max
-0.016311	-0.0037	-0.0013	0.00216	0.01761

Coefficients:

	Estimate	Std. Error	t value	Pr(>|t|)	
(Intercept)	0.0059591	0.00239	2.488	0.01905	*
Time	0.0009669	0.00023	4.18	0.00026	***

Signif.	codes:	0	'***'	0.001	'**'	0.01	'*'
		0.05	'.'	0.1	''	1	

Residual standard error:	0.007487	on 28 degrees of freedom
MultipleR-squared:	0.3842	
Adjusted R-squared:	0.3622	
F-statistic:	17.47	on 1 and 28 DF
p-value:	0.000259	

Tukey.HSD multiple comparison on treatments slope

$`50microM_TR`

$statistics

MSerror	Df	Mean	CV
0.009853	82	0.20854	47.59957

$parameters

test	name.t	ntr	StudentizedRange	alpha
Tukey	Treatment	3	3.375734	0.05

$means

	Value	std	r	Min	Max	Q25	Q50	Q75
C	0.167755	0.100018	30	-0.01169471	0.41446	0.09723	0.14815	0.21425
Fe	0.220569	0.10399	29	0.02248622	0.48588	0.16951	0.21314	0.25669
P	0.238702	0.121957	29	0.08516335	0.62276	0.15795	0.20172	0.29531

$groups

	Value	groups
P	0.238702	a
Fe	0.220569	ab
C	0.167755	b

$`50microM_TS`

$statistics

MSerror	Df	Mean	CV	MSD
1.06E-05	84	0.00209	155.454	0.002

$parameters

test	name.t	ntr	StudentizedRange	alpha
Tukey	Treatment	3	3.374254	0.05

$means

	Value	std	r	Min	Max	Q25	Q50	Q75
C	0.001102	0.004619	30	-0.008607855	0.01162	-0.00078	0.00154	0.0038
Fe	0.00321	0.003162	30	-0.005320243	0.00993	0.00128	0.00287	0.00502
P	0.001959	0.003423	30	-0.004952103	0.00913	-0.00053	0.00235	0.00405

$groups

	Value	groups
Fe	0.00321	a
P	0.001959	ab
C	0.001102	b

$`500microM_TR`

$statistics

MSerror	Df	Mean	CV
0.122771	83	0.75877	46.17816

$parameters

	test	name.t	ntr	StudentizedRange	alpha
	Tukey	Treatment	3	3.374985	0.05

$means

	Value	std	r	Min	Max	Q25	Q50	Q75
C	0.669289	0.359314	30	0.2796703	1.42114	0.36788	0.55255	0.96102
Fe	0.777436	0.468033	29	0.2274075	1.96371	0.44179	0.59445	1.01678
P	0.830215	0.429333	30	0.1692187	1.65114	0.48829	0.76778	1.05098

$groups

	Value	groups
P	0.830215	a
Fe	0.777436	a
C	0.669289	a

$`500microM_TS`

$statistics

MSerror	Df	Mean	CV	MSD
3.28E-05	84	0.00988	57.94127	0.00353

$parameters

test	name.t	ntr	StudentizedRange	alpha
Tukey	Treatment	3	3.374254	0.05

$means

	Value	std	r	Min	Max	Q25	Q50	Q75
C	0.006719	0.004885	30	-0.005698787	0.01519	0.00495	0.00667	0.00881
Fe	0.014178	0.009375	30	0.001878568	0.0397	0.00629	0.01397	0.01761
P	0.008738	0.005259	30	-0.0000664	0.02057	0.0049	0.00767	0.01272

$groups

	Value	groups
Fe	0.014178	a
P	0.008738	b
C	0.006719	b
