# Supplementary material for: Exploring glycine root uptake dynamics in phosphorus and iron deficient tomato plants during the initial stages of plant development
Source: BMC Plant Biol. 2024 Jun 3;24:495. doi: 10.1186/s12870-024-05120-6 (PMC11145798; doi:10.1186/s12870-024-05120-6)
Supplement: Supplementary file 6 — Supplementary Material 6. [file 12870_2024_5120_MOESM6_ESM.pdf]

Table S5: Table summarizing the statistical analyses performed on the fresh weight (mg) (Fig. S1). The table is divided in six sections: summary table for number of replicates (N), mean relative abundance, standard deviation (sd) and standard error (se), Two-Way ANOVA, One-Way ANOVA on treatments, Tukey.HSD multiple comparison on treatments, One-Way ANOVA on time (plant development) and Tukey.HSD multiple comparison on time (plant development). C = Control condition; -P = phosphorus deficiency; -Fe = iron deficiency.

Summary table

Treatment	Time	Glycine Concentration (µM)	Tissue	N	Fresh weight (mg)	sd	se
C	0	0	Root	5	15.60	6.99	3.12
C	0	0	Shoot	5	128.40	21.64	9.68
C	0	500	Root	5	13.80	2.49	1.11
C	0	500	Shoot	5	116.40	22.47	10.05
C	0	50	Root	5	16.00	3.46	1.55
C	0	50	Shoot	5	124.60	19.27	8.62
C	3	0	Root	5	34.40	9.96	4.46
C	3	0	Shoot	5	270.80	46.31	20.71
C	3	500	Root	5	43.00	12.98	5.81
C	3	500	Shoot	5	326.40	120.50	53.89
C	3	50	Root	5	38.40	13.24	5.92
C	3	50	Shoot	5	255.80	59.57	26.64
C	7	0	Root	5	129.60	44.53	19.91
C	7	0	Shoot	5	1061.80	295.55	132.18
C	7	500	Root	5	95.80	28.20	12.61
C	7	500	Shoot	5	887.20	299.77	134.06
C	7	50	Root	5	75.80	18.71	8.37
C	7	50	Shoot	5	409.00	28.29	12.65
C	10	0	Root	5	285.60	117.60	52.59
C	10	0	Shoot	5	1995.00	684.11	305.94
C	10	500	Root	5	250.20	75.28	33.67
C	10	500	Shoot	5	1981.80	633.45	283.29
C	10	50	Root	5	211.80	62.21	27.82
C	10	50	Shoot	5	1698.40	402.06	179.81
C	14	0	Root	5	783.20	65.81	29.43
C	14	0	Shoot	5	5962.60	706.17	315.81
C	14	500	Root	5	738.00	189.83	84.89
C	14	500	Shoot	5	5476.80	756.49	338.31
C	14	50	Root	5	588.80	277.53	124.11
C	14	50	Shoot	5	4972.20	1809.70	809.32
C	17	0	Root	5	1353.40	484.47	216.66
C	17	0	Shoot	5	8426.40	2861.02	1279.49
C	17	500	Root	5	1319.60	620.71	277.59
C	17	500	Shoot	5	9509.80	2829.87	1265.56
C	17	50	Root	5	1318.20	601.33	268.92
C	17	50	Shoot	5	8931.60	3261.46	1458.57
Fe	0	0	Root	5	17.80	5.07	2.27
Fe	0	0	Shoot	5	120.00	28.18	12.60
Fe	0	500	Root	5	11.60	5.55	2.48
Fe	0	500	Shoot	5	118.60	22.73	10.17

Fe	0	50	Root	5	14.80	4.97	2.22
Fe	0	50	Shoot	5	86.80	24.52	10.97
Fe	3	0	Root	5	36.40	15.18	6.79
Fe	3	0	Shoot	5	232.20	36.77	16.45
Fe	3	500	Root	5	49.80	21.00	9.39
Fe	3	500	Shoot	5	227.40	61.00	27.28
Fe	3	50	Root	5	34.60	18.15	8.12
Fe	3	50	Shoot	5	258.40	63.43	28.36
Fe	7	0	Root	5	74.20	50.13	22.42
Fe	7	0	Shoot	5	467.20	204.54	91.47
Fe	7	500	Root	5	96.60	23.93	10.70
Fe	7	500	Shoot	5	524.20	112.31	50.23
Fe	7	50	Root	5	89.20	35.74	15.99
Fe	7	50	Shoot	5	473.60	135.45	60.57
Fe	10	0	Root	5	157.00	35.59	15.92
Fe	10	0	Shoot	5	905.00	337.95	151.13
Fe	10	500	Root	5	160.40	59.11	26.44
Fe	10	500	Shoot	5	819.00	216.37	96.76
Fe	10	50	Root	5	219.20	66.21	29.61
Fe	10	50	Shoot	5	1156.20	368.43	164.77
Fe	14	0	Root	5	227.20	67.61	30.24
Fe	14	0	Shoot	5	1206.80	379.46	169.70
Fe	14	500	Root	5	322.00	106.16	47.48
Fe	14	500	Shoot	5	2114.20	487.45	217.99
Fe	14	50	Root	5	293.80	132.71	59.35
Fe	14	50	Shoot	5	1623.60	546.99	244.62
Fe	17	0	Root	5	542.80	326.49	146.01
Fe	17	0	Shoot	5	3003.00	1524.35	681.71
Fe	17	500	Root	4	419.50	106.02	53.01
Fe	17	500	Shoot	5	2179.00	767.74	343.34
Fe	17	50	Root	5	607.20	321.25	143.67
Fe	17	50	Shoot	5	3414.60	518.67	231.95
P	0	0	Root	5	14.60	3.51	1.57
P	0	0	Shoot	5	126.20	35.54	15.89
P	0	500	Root	5	15.80	3.42	1.53
P	0	500	Shoot	5	118.00	21.27	9.51
P	0	50	Root	5	16.40	2.88	1.29
P	0	50	Shoot	5	125.20	10.99	4.91
P	3	0	Root	5	40.20	18.14	8.11
P	3	0	Shoot	5	236.60	60.87	27.22
P	3	500	Root	5	48.20	16.39	7.33
P	3	500	Shoot	5	270.80	86.08	38.50
P	3	50	Root	5	34.00	10.07	4.51
P	3	50	Shoot	5	268.00	41.67	18.64
P	7	0	Root	5	103.40	18.26	8.16
P	7	0	Shoot	5	396.40	91.82	41.06
P	7	500	Root	5	82.20	27.40	12.25
P	7	500	Shoot	5	407.80	35.44	15.85
P	7	50	Root	5	111.20	29.58	13.23
P	7	50	Shoot	5	486.60	100.45	44.92

P	10	0	Root	5	138.20	56.21	25.14
P	10	0	Shoot	5	508.20	143.91	64.36
P	10	500	Root	5	193.20	84.31	37.70
P	10	500	Shoot	5	590.40	195.67	87.51
P	10	50	Root	5	214.20	61.62	27.56
P	10	50	Shoot	5	638.00	151.43	67.72
P	14	0	Root	5	235.60	42.55	19.03
P	14	0	Shoot	5	560.40	123.57	55.26
P	14	500	Root	5	257.60	112.95	50.51
P	14	500	Shoot	5	656.20	174.94	78.24
P	14	50	Root	5	219.00	91.99	41.14
P	14	50	Shoot	5	559.60	181.97	81.38
P	17	0	Root	5	249.80	113.50	50.76
P	17	0	Shoot	5	614.60	147.57	66.00
P	17	500	Root	5	285.20	70.31	31.44
P	17	500	Shoot	5	762.40	237.25	106.10
P	17	50	Root	5	292.40	82.72	36.99
P	17	50	Shoot	5	637.20	136.97	61.25

Two-Way ANOVA

Tissue	Gly Concentration (µM)	Statistical parameter	Treatment	Time	Treatment:Time	Residuals
		Df	2	5	10	72
**Root**	**0**	*F value*	37.16	51.24	12.74	
		P value	0.000	<2.20E	0.000	
		Df	2	5	10	71
**Root**	**50**	*F value*	12.60	34.77	7.08	
		P value	0.000	0.000	0.000	
		Df	2	5	10	72
**Root**	**500**	*F value*	23.68	37.77	9.58	
		P value	0.000	0.000	0.000	
		Df	2	5	10	72
**Shoot**	**0**	*F value*	81.94	53.38	21.99	
		P value	0.000	0.000	0.000	
		Df	2	5	10	72
**Shoot**	**50**	*F value*	49.27	48.70	18.44	
		P value	0.000	0.000	0.000	
		Df	2	5	10	72
**Shoot**	**500**	*F value*	99.34	69.06	31.36	
		P value	0.000	0.000	0.000	

One-Way ANOVA Treatment

Tissue	*Gly Concentration (µM)*	*Time*	*Statistical parameter*	*Treatment*	*Residuals*
**Root**	**0**	**0**	*Df*	2	12
			F value	0.46	
			P value	0.640	
**Root**	**0**	**3**	*Df*	2	12
			F value	0.20	
			P value	0.823	
**Root**	**0**	**7**	*Df*	2	12
			F value	2.39	
			P value	0.134	
**Root**	**0**	**10**	*Df*	2	12
			F value	5.29	
			P value	0.023	
**Root**	**0**	**14**	*Df*	2	12
			F value	142.13	
			P value	0.000	
**Root**	**0**	**17**	*Df*	2	12
			F value	13.84	
			P value	0.001	
**Root**	**50**	**0**	*Df*	2	12
			F value	0.23	
			P value	0.797	
**Root**	**50**	**3**	*Df*	2	12
			F value	0.14	
			P value	0.870	
**Root**	**50**	**7**	*Df*	2	12
			F value	1.91	
			P value	0.190	
**Root**	**50**	**10**	*Df*	2	12
			F value	0.02	
			P value	0.982	
**Root**	**50**	**14**	*Df*	2	12
			F value	5.56	
			P value	0.020	
**Root**	**50**	**17**	*Df*	2	12
			F value	8.78	
			P value	0.004	
**Root**	**500**	**0**	*Df*	2	12
			F value	1.36	
			P value	0.294	
**Root**	**500**	**3**	*Df*	2	12
			F value	0.22	
			P value	0.809	
**Root**	**500**	**7**	*Df*	2	12
			F value	0.46	
			P value	0.640	

**Root**	**500**	**10**	*Df*	2	12
			F value	1.90	
			P value	0.191	
**Root**	**500**	**14**	*Df*	2	12
			F value	16.98	
			P value	0.000	
**Root**	**500**	**17**	*Df*	2	11
			F value	10.67	
			P value	0.003	
**Shoot**	**0**	**0**	*Df*	2	12
			F value	0.11	
			P value	0.894	
**Shoot**	**0**	**3**	*Df*	2	12
			F value	0.93	
			P value	0.421	
**Shoot**	**0**	**7**	*Df*	2	12
			F value	14.56	
			P value	0.001	
**Shoot**	**0**	**10**	*Df*	2	12
			F value	14.75	
			P value	0.001	
**Shoot**	**0**	**14**	*Df*	2	12
			F value	198.42	
			P value	0.000	
**Shoot**	**0**	**17**	*Df*	2	12
			F value	22.82	
			P value	0.000	
**Shoot**	**50**	**0**	*Df*	2	12
			F value	6.64	
			P value	0.011	
**Shoot**	**50**	**3**	*Df*	2	12
			F value	0.07	
			P value	0.936	
**Shoot**	**50**	**7**	*Df*	2	12
			F value	0.89	
			P value	0.438	
**Shoot**	**50**	**10**	*Df*	2	12
			F value	13.17	
			P value	0.001	
**Shoot**	**50**	**14**	*Df*	2	12
			F value	22.05	
			P value	0.000	
**Shoot**	**50**	**17**	*Df*	2	12
			F value	24.47	
			P value	0.000	
**Shoot**	**500**	**0**	*Df*	2	12
			F value	0.01	
			P value	0.987	
**Shoot**	**500**	**3**	*Df*	2	12
			F value	1.44	

			P value	0.275	
			Df	2	12
**Shoot**	**500**	**7**	*F value*	9.04	
			P value	0.004	
			Df	2	12
**Shoot**	**500**	**10**	*F value*	17.17	
			P value	0.000	
			Df	2	12
**Shoot**	**500**	**14**	*F value*	109.08	
			P value	0.000	
			Df	2	12
**Shoot**	**500**	**17**	*F value*	38.21	
			P value	0.000	

Tukey.HSD multiple comparison on Treatment

$`0µM_TR`

$`0µM_TR`$`0`

	Value	groups
Fe	17.8	a
C	15.6	a
P	14.6	a

$`0µM_TR`$`3`

	Value	groups
P	40.2	a
Fe	36.4	a
C	34.4	a

$`0µM_TR`$`7`

	Value	groups
C	129.6	a
P	103.4	a
Fe	74.2	a

$`0µM_TR`$`10`

	Value	groups
C	285.6	a
Fe	157	ab
P	138.2	b

$`0µM_TR`$`14`

	Value	groups
C	783.2	a
P	235.6	b
Fe	227.2	b

$`0µM_TR`$`17`

	Value	groups
C	1353.4	a
Fe	542.8	b
P	249.8	b

$`0µM_TS`

$`0µM_TS`$`0`

	Value	groups
C	128.4	a
P	126.2	a
Fe	120	a

$`0µM_TS`$`3`

	Value	groups
C	270.8	a

P	236.6	a
Fe	232.2	a

$`0µM_TS`$`7`

	Value	groups
C	1061.8	a
Fe	467.2	b
P	396.4	b

$`0µM_TS`$`10`

	Value	groups
C	1995	a
Fe	905	b
P	508.2	b

$`0µM_TS`$`14`

	Value	groups
C	5962.6	a
Fe	1206.8	b
P	560.4	b

$`0µM_TS`$`17`

	Value	groups
C	8426.4	a
Fe	3003	b
P	614.6	b

$`50µM_TR`

$`50µM_TR`$`0`

	Value	groups
P	16.4	a
C	16	a
Fe	14.8	a

$`50µM_TR`$`3`

	Value	groups
C	38.4	a
Fe	34.6	a
P	34	a

$`50µM_TR`$`7`

	Value	groups
P	111.2	a
Fe	89.2	a
C	75.8	a

$`50µM_TR`$`10`

	Value	groups
Fe	219.2	a

P	214.2	a
C	211.8	a

$`50µM_TR`$`14`

	Value	groups
C	588.8	a
Fe	293.8	ab
P	219	b

$`50µM_TR`$`17`

	Value	groups
C	1318.2	a
Fe	607.2	b
P	292.4	b

$`50µM_TS`

$`50µM_TS`$`0`

	Value	groups
P	125.2	a
C	124.6	a
Fe	86.8	b

$`50µM_TS`$`3`

	Value	groups
P	268	a
Fe	258.4	a
C	255.8	a

$`50µM_TS`$`7`

	Value	groups
P	486.6	a
Fe	473.6	a
C	409	a

$`50µM_TS`$`10`

	Value	groups
C	1698.4	a
Fe	1156.2	ab
P	638	b

$`50µM_TS`$`14`

	Value	groups
C	4972.2	a
Fe	1623.6	b
P	559.6	b

$`50µM_TS`$`17`

	Value	groups
C	8931.6	a

Fe	3414.6	b
P	637.2	b

$`500µM_TR`

$`500µM_TR`$`0`

	Value	groups
P	15.8	a
C	13.8	a
Fe	11.6	a

$`500µM_TR`$`3`

	Value	groups
Fe	49.8	a
P	48.2	a
C	43	a

$`500µM_TR`$`7`

	Value	groups
Fe	96.6	a
C	95.8	a
P	82.2	a

$`500µM_TR`$`10`

	Value	groups
C	250.2	a
P	193.2	a
Fe	160.4	a

$`500µM_TR`$`14`

	Value	groups
C	738	a
Fe	322	b
P	257.6	b

$`500µM_TR`$`17`

	Value	groups
C	1319.6	a
Fe	419.5	b
P	285.2	b

$`500µM_TS`

$`500µM_TS`$`0`

	Value	groups
Fe	118.6	a
P	118	a
C	116.4	a

$`500µM_TS`$`3`

	Value	groups
C	326.4	a
P	270.8	a
Fe	227.4	a

$`500µM_TS`$`7`

	Value	groups
C	887.2	a
Fe	524.2	b
P	407.8	b

$`500µM_TS`$`10`

	Value	groups
C	1981.8	a
Fe	819	b
P	590.4	b

$`500µM_TS`$`14`

	Value	groups
C	5476.8	a
Fe	2114.2	b
P	656.2	c

$`500µM_TS`$`17`

	Value	groups
C	9509.8	a
Fe	2179	b
P	762.4	b

One-Way ANOVA Time

Tissue	*Gly Concentration (μM)*	*Treatment*	*Statistical parameter*	*Time*	*Residuals*
**Root**	**0**	**C**	*Df*	5	24
			F value	33.33	
			P value	0.000	
**Root**	**0**	**Fe**	*Df*	5	24
			F value	10.02	
			P value	0.000	
**Root**	**0**	**P**	*Df*	5	24
			F value	15.43	
			P value	0.000	
**Root**	**50**	**C**	*Df*	5	24
			F value	17.52	
			P value	0.000	
**Root**	**50**	**Fe**	*Df*	5	24
			F value	11.75	
			P value	0.000	
**Root**	**50**	**P**	*Df*	5	24
			F value	18.50	
			P value	0.000	
**Root**	**500**	**C**	*Df*	5	24
			F value	18.94	
			P value	0.000	
**Root**	**500**	**Fe**	*Df*	5	23
			F value	27.97	
			P value	0.000	
**Root**	**500**	**P**	*Df*	5	24
			F value	15.01	
			P value	0.000	
**Shoot**	**0**	**C**	*Df*	5	24
			F value	38.10	
			P value	0.000	
**Shoot**	**0**	**Fe**	*Df*	5	24
			F value	13.05	
			P value	0.000	
**Shoot**	**0**	**P**	*Df*	5	24
			F value	15.59	
			P value	0.000	
**Shoot**	**50**	**C**	*Df*	5	24
			F value	26.79	
			P value	0.000	
**Shoot**	**50**	**Fe**	*Df*	5	24
			F value	63.83	
			P value	0.000	
**Shoot**	**50**	**P**	*Df*	5	24
			F value	15.38	
			P value	0.000	

**Shoot**	**500**	**C**	*Df*	5	24
			F value	45.87	
			P value	0.000	
**Shoot**	**500**	**Fe**	*Df*	5	24
			F value	28.73	
			P value	0.000	
**Shoot**	**500**	**P**	*Df*	5	24
			F value	13.50	
			P value	0.000	

Tukey.HSD multiple comparison on Time

$`0µM_TR`
$`0µM_TR`$C

	Value	groups
17	1353.4	a
14	783.2	b
10	285.6	c
7	129.6	c
3	34.4	c
0	15.6	c

$`0µM_TR`$Fe

	Value	groups
17	542.8	a
14	227.2	b
10	157	b
7	74.2	b
3	36.4	b
0	17.8	b

$`0µM_TR`$P

	Value	groups
17	249.8	a
14	235.6	ab
10	138.2	bc
7	103.4	cd
3	40.2	cd
0	14.6	d

$`0µM_TS`
$`0µM_TS`$C

	Value	groups
17	8426.4	a
14	5962.6	b
10	1995	c
7	1061.8	c
3	270.8	c
0	128.4	c

$`0µM_TS`$Fe

	Value	groups
17	3003	a
14	1206.8	b
10	905	b
7	467.2	b
3	232.2	b
0	120	b

$`0µM_TS`$P

	Value	groups
17	614.6	a
14	560.4	ab
10	508.2	ab
7	396.4	bc
3	236.6	cd
0	126.2	d

$`50µM_TR`
$`50µM_TR`$C

	Value	groups
17	1318.2	a
14	588.8	b
10	211.8	bc
7	75.8	bc
3	38.4	c
0	16	c

$`50µM_TR`$Fe

	Value	groups
17	607.2	a
14	293.8	b
10	219.2	b
7	89.2	b
3	34.6	b
0	14.8	b

$`50µM_TR`$P

	Value	groups
17	292.4	a
14	219	ab
10	214.2	ab
7	111.2	bc
3	34	c
0	16.4	c

$`50µM_TS`
$`50µM_TS`$C

	Value	groups
17	8931.6	a
14	4972.2	b
10	1698.4	c
7	409	c
3	255.8	c
0	124.6	c

$`50µM_TS`$Fe

	Value	groups
17	3414.6	a
14	1623.6	b
10	1156.2	b
7	473.6	c
3	258.4	c
0	86.8	c

$`50µM_TS`$P

	Value	groups
10	638	a
17	637.2	a
14	559.6	a
7	486.6	ab
3	268	bc
0	125.2	c

$`500µM_TR`

$`500µM_TR`$C

	Value	groups
17	1319.6	a
14	738	b
10	250.2	bc
7	95.8	c
3	43	c
0	13.8	c

$`500µM_TR`$Fe

	Value	groups
17	419.5	a
14	322	a
10	160.4	b
7	96.6	bc
3	49.8	bc
0	11.6	c

$`500µM_TR`$P

	Value	groups
17	285.2	a
14	257.6	a
10	193.2	ab
7	82.2	bc
3	48.2	c
0	15.8	c

$`500µM_TS`

$`500µM_TS`$C

	Value	groups

17	9509.8	a
14	5476.8	b
10	1981.8	c
7	887.2	c
3	326.4	c
0	116.4	c

$`500µM_TS`$Fe

	Value	groups
17	2179	a
14	2114.2	a
10	819	b
7	524.2	b
3	227.4	b
0	118.6	b

$`500µM_TS`$P

	Value	groups
17	762.4	a
14	656.2	ab
10	590.4	ab
7	407.8	bc
3	270.8	c
0	118	c
