# Supplementary material for: Exploring glycine root uptake dynamics in phosphorus and iron deficient tomato plants during the initial stages of plant development
Source: BMC Plant Biol. 2024 Jun 3;24:495. doi: 10.1186/s12870-024-05120-6 (PMC11145798; doi:10.1186/s12870-024-05120-6)
Supplement: Supplementary file 7 — Supplementary Material 7. [file 12870_2024_5120_MOESM7_ESM.pdf]

Table S6: Table summarizing the statistical analyses performed on the δ13C (Fig. S2). The table is divided in six sections: summary table for number of replicates (N), mean relative abundance, standard deviation (sd) and standard error (se), Two-Way ANOVA, One-Way ANOVA on treatments, Tukey.HSD multiple comparison on treatments, One-Way ANOVA on time (plant development) and Tukey.HSD multiple comparison on time (plant development). C = Control condition; -P = phosphorus deficiency; -Fe = iron deficiency.

Summary table

Treatment	Time	Glycine Concentration (µM)	Tissue	N	$\delta^{13}C$	sd	se
C	0	0	Root	5	-31.6449	0.651246	0.291246
C	0	0	Shoot	5	-35.7569	0.286881	0.128297
C	0	50	Root	5	16.11359	18.54975	8.295699
C	0	50	Shoot	5	-34.5851	0.77838	0.348102
C	0	500	Root	5	143.5857	27.87963	12.46815
C	0	500	Shoot	5	-33.779	0.577301	0.258177
C	3	0	Root	5	-33.5574	1.795956	0.803176
C	3	0	Shoot	5	-37.2279	0.186417	0.083368
C	3	50	Root	5	25.88849	16.76066	7.495596
C	3	50	Shoot	5	-36.9061	0.427417	0.191146
C	3	500	Root	5	167.4422	101.6987	45.48105
C	3	500	Shoot	5	-36.1495	0.165866	0.074177
C	7	0	Root	5	-36.2204	0.273114	0.12214
C	7	0	Shoot	5	-37.3571	0.37989	0.169892
C	7	50	Root	5	-9.06423	10.25142	4.584575
C	7	50	Shoot	5	-37.1552	0.481184	0.215192
C	7	500	Root	5	101.0002	76.8181	34.3541
C	7	500	Shoot	5	-35.0744	0.800124	0.357826
C	10	0	Root	5	-35.8565	0.532229	0.23802
C	10	0	Shoot	5	-38.1493	0.303445	0.135705
C	10	50	Root	5	-24.1315	8.712368	3.896289
C	10	50	Shoot	5	-37.6858	0.464036	0.207523
C	10	500	Root	5	35.0919	45.16136	20.19677
C	10	500	Shoot	5	-36.8624	0.383597	0.17155
C	14	0	Root	5	-36.1422	0.344972	0.154276
C	14	0	Shoot	5	-37.1836	0.29724	0.13293
C	14	50	Root	5	-11.0466	6.021335	2.692823
C	14	50	Shoot	5	-37.4357	0.48784	0.218169
C	14	500	Root	5	49.60353	17.85686	7.985829
C	14	500	Shoot	5	-35.8965	0.333511	0.149151
C	17	0	Root	5	-36.4368	0.305286	0.136528
C	17	0	Shoot	5	-36.8035	0.632121	0.282693
C	17	50	Root	5	-20.8682	7.764783	3.472516
C	17	50	Shoot	5	-37.4242	1.163403	0.52029
C	17	500	Root	5	36.82872	25.49945	11.4037
C	17	500	Shoot	5	-36.9354	0.726678	0.32498
Fe	0	0	Root	5	-32.1112	1.209237	0.540787
Fe	0	0	Shoot	5	-35.3523	0.603912	0.270078
Fe	0	50	Root	5	6.047048	10.67352	4.773343
Fe	0	50	Shoot	5	-35.0747	0.14516	0.064918

Fe	0	500	Root	5	137.2669	76.2931	34.11931
Fe	0	500	Shoot	5	-34.7192	0.330044	0.1476
Fe	3	0	Root	5	-34.7896	0.258629	0.115662
Fe	3	0	Shoot	5	-37.1118	0.202763	0.090679
Fe	3	50	Root	5	31.32636	36.12541	16.15578
Fe	3	50	Shoot	5	-36.7242	0.409274	0.183033
Fe	3	500	Root	5	264.638	131.8043	58.94467
Fe	3	500	Shoot	5	-35.7689	0.341388	0.152673
Fe	7	0	Root	5	-36.6943	0.424141	0.189681
Fe	7	0	Shoot	5	-37.9073	0.431336	0.192899
Fe	7	50	Root	5	-6.68798	11.39778	5.097241
Fe	7	50	Shoot	5	-37.055	0.216031	0.096612
Fe	7	500	Root	5	126.3864	72.91654	32.60927
Fe	7	500	Shoot	5	-34.0981	1.440248	0.644098
Fe	10	0	Root	5	-37.558	0.302292	0.135189
Fe	10	0	Shoot	5	-38.163	0.208282	0.093147
Fe	10	50	Root	5	7.028643	9.124056	4.080402
Fe	10	50	Shoot	5	-36.9006	0.681837	0.304927
Fe	10	500	Root	5	41.27536	25.36446	11.34333
Fe	10	500	Shoot	5	-34.8578	1.79877	0.804435
Fe	14	0	Root	5	-36.5993	0.433136	0.193704
Fe	14	0	Shoot	5	-37.3993	0.345932	0.154706
Fe	14	50	Root	5	0.900106	7.925025	3.544179
Fe	14	50	Shoot	5	-36.977	0.663912	0.29691
Fe	14	500	Root	5	54.12514	31.69487	14.17438
Fe	14	500	Shoot	5	-34.3476	0.513379	0.22959
Fe	17	0	Root	5	-37.3611	0.361048	0.161466
Fe	17	0	Shoot	5	-38.0601	0.604146	0.270182
Fe	17	50	Root	4	-18.8718	13.87368	6.936838
Fe	17	50	Shoot	5	-37.3414	1.042172	0.466074
Fe	17	500	Root	4	48.61308	37.99865	18.99933
Fe	17	500	Shoot	5	-33.3048	3.04227	1.360544
P	0	0	Root	5	-33.068	1.508801	0.674756
P	0	0	Shoot	5	-35.4877	0.687539	0.307477
P	0	50	Root	5	16.68805	18.93748	8.469097
P	0	50	Shoot	5	-34.6591	0.595005	0.266094
P	0	500	Root	5	119.6185	83.35367	37.2769
P	0	500	Shoot	5	-34.6354	0.585433	0.261814
P	3	0	Root	5	-35.3387	0.934021	0.417707
P	3	0	Shoot	5	-37.3684	0.22425	0.100288
P	3	50	Root	5	11.36782	26.54023	11.86915
P	3	50	Shoot	5	-36.6655	0.169722	0.075902
P	3	500	Root	5	262.9497	51.51041	23.03616
P	3	500	Shoot	5	-35.8051	0.958029	0.428444
P	7	0	Root	5	-35.6888	0.238615	0.106712
P	7	0	Shoot	5	-37.0282	0.495208	0.221464
P	7	50	Root	4	25.9059	33.40948	16.70474
P	7	50	Shoot	5	-36.5341	0.423638	0.189457
P	7	500	Root	5	118.0302	40.92822	18.30366
P	7	500	Shoot	5	-35.0606	0.614874	0.27498

P	10	0	Root	5	-34.4681	0.713371	0.319029
P	10	0	Shoot	5	-36.5772	0.591242	0.264412
P	10	50	Root	5	1.685796	9.616169	4.300482
P	10	50	Shoot	5	-36.6734	0.085116	0.038065
P	10	500	Root	5	129.8557	32.51935	14.5431
P	10	500	Shoot	5	-35.7369	0.769782	0.344257
P	14	0	Root	5	-34.9739	0.625794	0.279864
P	14	0	Shoot	5	-36.6758	0.226323	0.101215
P	14	50	Root	5	4.403145	9.980756	4.46353
P	14	50	Shoot	5	-36.0905	0.66405	0.296972
P	14	500	Root	5	50.53493	22.5626	10.0903
P	14	500	Shoot	5	-33.8109	0.397772	0.177889
P	17	0	Root	5	-35.0593	1.529929	0.684205
P	17	0	Shoot	5	-35.2798	0.819452	0.36647
P	17	50	Root	5	-1.8812	25.91048	11.58752
P	17	50	Shoot	5	-35.6529	0.663623	0.296781
P	17	500	Root	5	45.64438	40.91574	18.29807
P	17	500	Shoot	5	-33.7368	0.671984	0.30052

Two-Way ANOVA

Tissue	*Gly Concentration (μM)*	*Statistical parameter*	*Treatment*	*Time*	*Treatment:Time*	*Residuals*
**Root**	**0**	*Df*	2	5	10	72
		F value	14.02	51.62	5.72	
		P value	0.000	0.000	0.000	
**Root**	**50**	*Df*	2	5	10	70
		F value	4.02	7.98	2.13	
		P value	0.022	0.000	0.033	
**Root**	**500**	*Df*	2	5	10	71
		F value	2.32	19.91	1.25	
		P value	0.106	0.000	0.278	
**Shoot**	**0**	*Df*	2	5	10	72
		F value	32.81	40.60	8.42	
		P value	0.000	0.000	0.000	
**Shoot**	**50**	*Df*	2	5	10	72
		F value	15.31	31.29	2.32	
		P value	0.000	0.000	0.020	
**Shoot**	**500**	*Df*	2	5	10	72
		F value	12.14	5.92	3.74	
		P value	0.000	0.000	0.000	

One-Way ANOVA Treatment

Tissue	*Gly Concentration (μM)*	*Time*	*Statistical parameter*	*Treatment*	*Residuals*
**Root**	**0**	**0**	*Df*	2	12
			F value	1.90	
			P value	0.192	
**Root**	**0**	**3**	*Df*	2	12
			F value	3.00	
			P value	0.088	
**Root**	**0**	**7**	*Df*	2	12
			F value	12.19	
			P value	0.001	
**Root**	**0**	**10**	*Df*	2	12
			F value	40.66	
			P value	0.000	
**Root**	**0**	**14**	*Df*	2	12
			F value	15.10	
			P value	0.001	
**Root**	**0**	**17**	*Df*	2	12
			F value	7.85	
			P value	0.007	
**Root**	**50**	**0**	*Df*	2	12
			F value	0.66	
			P value	0.536	
**Root**	**50**	**3**	*Df*	2	12
			F value	0.70	
			P value	0.517	
**Root**	**50**	**7**	*Df*	2	11
			F value	4.20	
			P value	0.044	
**Root**	**50**	**10**	*Df*	2	12
			F value	16.55	
			P value	0.000	
**Root**	**50**	**14**	*Df*	2	12
			F value	4.95	
			P value	0.027	
**Root**	**50**	**17**	*Df*	2	11
			F value	1.67	
			P value	0.233	
**Root**	**500**	**0**	*Df*	2	12
			F value	0.17	
			P value	0.845	
**Root**	**500**	**3**	*Df*	2	12
			F value	1.53	
			P value	0.256	
**Root**	**500**	**7**	*Df*	2	12
			F value	0.19	
			P value	0.826	

**Root**	**500**	**10**	*Df*	2	12
			F value	11.27	
			P value	0.002	
**Root**	**500**	**14**	*Df*	2	12
			F value	0.05	
			P value	0.955	
**Root**	**500**	**17**	*Df*	2	11
			F value	0.14	
			P value	0.870	
**Shoot**	**0**	**0**	*Df*	2	12
			F value	0.69	
			P value	0.520	
**Shoot**	**0**	**3**	*Df*	2	12
			F value	1.96	
			P value	0.183	
**Shoot**	**0**	**7**	*Df*	2	12
			F value	5.14	
			P value	0.024	
**Shoot**	**0**	**10**	*Df*	2	12
			F value	25.70	
			P value	0.000	
**Shoot**	**0**	**14**	*Df*	2	12
			F value	7.98	
			P value	0.006	
**Shoot**	**0**	**17**	*Df*	2	12
			F value	20.25	
			P value	0.000	
**Shoot**	**50**	**0**	*Df*	2	12
			F value	1.07	
			P value	0.375	
**Shoot**	**50**	**3**	*Df*	2	12
			F value	0.62	
			P value	0.553	
**Shoot**	**50**	**7**	*Df*	2	12
			F value	3.64	
			P value	0.058	
**Shoot**	**50**	**10**	*Df*	2	12
			F value	6.16	
			P value	0.014	
**Shoot**	**50**	**14**	*Df*	2	12
			F value	6.26	
			P value	0.014	
**Shoot**	**50**	**17**	*Df*	2	12
			F value	5.20	
			P value	0.024	
**Shoot**	**500**	**0**	*Df*	2	12
			F value	5.17	
			P value	0.024	
**Shoot**	**500**	**3**	*Df*	2	12
			F value	0.62	

			P value	0.553	
**Shoot**	**500**	**7**	*Df*	2	12
			F value	1.52	
			P value	0.258	
**Shoot**	**500**	**10**	*Df*	2	12
			F value	3.81	
			P value	0.052	
**Shoot**	**500**	**14**	*Df*	2	12
			F value	33.01	
			P value	0.000	
**Shoot**	**500**	**17**	*Df*	2	12
			F value	5.76	
			P value	0.018	

Tukey.HSD multiple comparison on treatment

$`0µM_TR`

$`0µM_TR`$`0`

	Value	groups
C	-31.6449	a
Fe	-32.1112	a
P	-33.068	a

$`0µM_TR`$`3`

	Value	groups
C	-33.5574	a
Fe	-34.7896	a
P	-35.3387	a

$`0µM_TR`$`7`

	Value	groups
P	-35.6888	a
C	-36.2204	ab
Fe	-36.6944	b

$`0µM_TR`$`10`

	Value	groups
P	-34.4681	a
C	-35.8565	b
Fe	-37.5581	c

$`0µM_TR`$`14`

	Value	groups
P	-34.9739	a
C	-36.1423	b
Fe	-36.5994	b

$`0µM_TR`$`17`

	Value	groups
P	-35.0593	a
C	-36.4368	ab
Fe	-37.3611	b

$`0µM_TS`

$`0µM_TS`$`0`

	Value	groups
Fe	-35.3523	a
P	-35.4877	a
C	-35.7569	a

$`0µM_TS`$`3`

	Value	groups
Fe	-37.1118	a

C	-37.2279	a
P	-37.3684	a

$`0µM_TS`$`7`

	Value	groups
P	-37.0283	a
C	-37.3571	ab
Fe	-37.9073	b

$`0µM_TS`$`10`

	Value	groups
P	-36.5772	a
C	-38.1493	b
Fe	-38.163	b

$`0µM_TS`$`14`

	Value	groups
P	-36.6758	a
C	-37.1836	b
Fe	-37.3993	b

$`0µM_TS`$`17`

	Value	groups
P	-35.2798	a
C	-36.8035	b
Fe	-38.0601	c

$`50µM_TR`

$`50µM_TR`$`0`

	Value	groups
P	16.68805	a
C	16.11359	a
Fe	6.047048	a

$`50µM_TR`$`3`

	Value	groups
Fe	31.32636	a
C	25.88849	a
P	11.36782	a

$`50µM_TR`$`7`

	Value	groups
P	25.9059	a
Fe	-6.68798	a
C	-9.06423	a

$`50µM_TR`$`10`

	Value	groups
Fe	7.028643	a

P	1.685796	a
C	-24.1315	b

$`50µM_TR`$`14`

	Value	groups
P	4.403145	a
Fe	0.900106	ab
C	-11.0466	b

$`50µM_TR`$`17`

	Value	groups
P	-1.8812	a
Fe	-18.8718	a
C	-20.8682	a

$`50µM_TS`

$`50µM_TS`$`0`

	Value	groups
C	-34.5851	a
P	-34.6592	a
Fe	-35.0747	a

$`50µM_TS`$`3`

	Value	groups
P	-36.6655	a
Fe	-36.7242	a
C	-36.9061	a

$`50µM_TS`$`7`

	Value	groups
P	-36.5341	a
Fe	-37.055	a
C	-37.1552	a

$`50µM_TS`$`10`

	Value	groups
P	-36.6734	a
Fe	-36.9006	ab
C	-37.6858	b

$`50µM_TS`$`14`

	Value	groups
P	-36.0905	a
Fe	-36.977	ab
C	-37.4357	b

$`50µM_TS`$`17`

	Value	groups
P	-35.6529	a

Fe	-37.3414	b
C	-37.4242	b

$`500µM_TR`

$`500µM_TR`$`0`

	Value	groups
C	143.5857	a
Fe	137.2669	a
P	119.6185	a

$`500µM_TR`$`3`

	Value	groups
Fe	264.638	a
P	262.9497	a
C	167.4422	a

$`500µM_TR`$`7`

	Value	groups
Fe	126.3864	a
P	118.0302	a
C	101.0002	a

$`500µM_TR`$`10`

	Value	groups
P	129.8557	a
Fe	41.27536	b
C	35.0919	b

$`500µM_TR`$`14`

	Value	groups
Fe	54.12514	a
P	50.53493	a
C	49.60353	a

$`500µM_TR`$`17`

	Value	groups
Fe	48.61308	a
P	45.64438	a
C	36.82872	a

$`500µM_TS`

$`500µM_TS`$`0`

	Value	groups
C	-33.779	a
P	-34.6354	ab
Fe	-34.7192	b

$`500µM_TS`$`3`

	Value	groups
Fe	-35.7689	a
P	-35.8051	a
C	-36.1495	a

$`500µM_TS`$`7`

	Value	groups
Fe	-34.0981	a
P	-35.0606	a
C	-35.0744	a

$`500µM_TS`$`10`

	Value	groups
Fe	-34.8578	a
P	-35.737	ab
C	-36.8624	b

$`500µM_TS`$`14`

	Value	groups
P	-33.8109	a
Fe	-34.3476	a
C	-35.8965	b

$`500µM_TS`$`17`

	Value	groups
Fe	-33.3048	a
P	-33.7368	a
C	-36.9354	b

One-Way ANOVA Time

Tissue	*Gly Concentration (μM)*	*Treatment*	*Statistical parameter*	*Time*	*Residuals*
**Root**	**0**	**C**	*Df*	5	24
			F value	26.91	
			P value	0.000	
**Root**	**0**	**Fe**	*Df*	5	24
			F value	61.12	
			P value	0.000	
**Root**	**0**	**P**	*Df*	5	24
			F value	3.98	
			P value	0.009	
**Root**	**50**	**C**	*Df*	5	24
			F value	13.71	
			P value	0.000	
**Root**	**50**	**Fe**	*Df*	5	23
			F value	4.07	
			P value	0.009	
**Root**	**50**	**P**	*Df*	5	23
			F value	1.01	
			P value	0.433	
**Root**	**500**	**C**	*Df*	5	24
			F value	4.93	
			P value	0.003	
**Root**	**500**	**Fe**	*Df*	5	23
			F value	6.57	
			P value	0.001	
**Root**	**500**	**P**	*Df*	5	24
			F value	12.76	
			P value	0.000	
**Shoot**	**0**	**C**	*Df*	5	24
			F value	21.96	
			P value	0.000	
**Shoot**	**0**	**Fe**	*Df*	5	24
			F value	29.60	
			P value	0.000	
**Shoot**	**0**	**P**	*Df*	5	24
			F value	11.50	
			P value	0.000	
**Shoot**	**50**	**C**	*Df*	5	24
			F value	14.00	
			P value	0.000	
**Shoot**	**50**	**Fe**	*Df*	5	24
			F value	8.87	
			P value	0.000	
**Shoot**	**50**	**P**	*Df*	5	24
			F value	12.80	
			P value	0.000	

			Df	5	24
**Shoot**	**500**	**C**	*F value*	24.03	
			P value	0.000	
			Df	5	24
**Shoot**	**500**	**Fe**	*F value*	1.35	
			P value	0.276	
			Df	5	24
**Shoot**	**500**	**P**	*F value*	8.64	
			P value	0.000	

Tukey.HSD multiple comparison on time

$`0µM_TR`

$`0µM_TR`$C

	Value	groups
0	-31.6449	a
3	-33.5574	b
10	-35.8565	c
14	-36.1423	c
7	-36.2204	c
17	-36.4368	c

$`0µM_TR`$Fe

	Value	groups
0	-32.1112	a
3	-34.7896	b
14	-36.5994	c
7	-36.6944	c
17	-37.3611	c
10	-37.5581	c

$`0µM_TR`$P

	Value	groups
0	-33.068	a
10	-34.4681	ab
14	-34.9739	ab
17	-35.0593	ab
3	-35.3387	b
7	-35.6888	b

$`0µM_TS`

$`0µM_TS`$C

	Value	groups
0	-35.7569	a
17	-36.8035	b
14	-37.1836	b
3	-37.2279	b
7	-37.3571	b
10	-38.1493	c

$`0µM_TS`$Fe

	Value	groups
0	-35.3523	a
3	-37.1118	b
14	-37.3993	bc
7	-37.9073	bc
17	-38.0601	c
10	-38.163	c

$`0µM_TS`$P

	Value	groups
17	-35.2798	a
0	-35.4877	a
10	-36.5772	b
14	-36.6758	b
7	-37.0283	b
3	-37.3684	b

$`50µM_TR`

$`50µM_TR`$C

	Value	groups
3	25.88849	a
0	16.11359	a
7	-9.06423	b
14	-11.0466	b
17	-20.8682	b
10	-24.1315	b

$`50µM_TR`$Fe

	Value	groups
3	31.32636	a
10	7.028643	ab
0	6.047048	ab
14	0.900106	ab
7	-6.68798	b
17	-18.8718	b

$`50µM_TR`$P

	Value	groups
7	25.9059	a
0	16.68805	a
3	11.36782	a
14	4.403145	a
10	1.685796	a
17	-1.8812	a

$`50µM_TS`

$`50µM_TS`$C

	Value	groups
0	-34.5851	a
3	-36.9061	b
7	-37.1552	b
17	-37.4242	b
14	-37.4357	b
10	-37.6858	b

$`50µM_TS`$Fe

	Value	groups
0	-35.0747	a
3	-36.7242	b
10	-36.9006	b
14	-36.977	b
7	-37.055	b
17	-37.3414	b

$`50µM_TS`$P

	Value	groups
0	-34.6592	a
17	-35.6529	b
14	-36.0905	bc
7	-36.5341	bc
3	-36.6655	c
10	-36.6734	c

$`500µM_TR`

$`500µM_TR`$C

	Value	groups
3	167.4422	a
0	143.5857	ab
7	101.0003	ab
14	49.60353	b
17	36.82872	b
10	35.0919	b

$`500µM_TR`$Fe

	Value	groups
3	264.638	a
0	137.267	ab
7	126.3864	ab
14	54.12514	b
17	48.61308	b
10	41.27536	b

$`500µM_TR`$P

	Value	groups
3	262.9498	a
10	129.8557	b
0	119.6186	b
7	118.0302	b
14	50.53493	b
17	45.64438	b

$`500µM_TS`

$`500µM_TS`$C

	Value	groups

0	-33.779	a
7	-35.0744	b
14	-35.8965	bc
3	-36.1495	c
10	-36.8624	c
17	-36.9354	c

$`500µM_TS`$Fe

	Value	groups
17	-33.3048	a
7	-34.0981	a
14	-34.3476	a
0	-34.7192	a
10	-34.8578	a
3	-35.7689	a

$`500µM_TS`$P

	Value	groups
17	-33.7368	a
14	-33.8109	a
0	-34.6354	ab
7	-35.0606	ab
10	-35.737	b
3	-35.8051	b
